# Interactions between Viral Regulatory Proteins Ensure an MOI-Independent Probability of Lysogeny during Infection by Bacteriophage P1

**DOI:** 10.1128/mBio.01013-21

**Published:** 2021-09-14

**Authors:** Kailun Zhang, Kiara Pankratz, Hau Duong, Matthew Theodore, Jingwen Guan, Anxiao (Andrew) Jiang, Yiruo Lin, Lanying Zeng

**Affiliations:** a Department of Biochemistry and Biophysics, Texas A&M University, College Station, Texas, USA; b Center for Phage Technology, Texas A&M University, College Station, Texas, USA; c Molecular and Environmental Plant Sciences, Texas A&M University, College Station, Texas, USA; d Department of Computer Science and Engineering, Texas A&M University, College Station, Texas, USA; National Cancer Institute

**Keywords:** bacteriophage P1, cell fate, gene regulatory network, infection, lysis-lysogeny, phage communication

## Abstract

Phage P1 is a temperate phage which makes the lytic or lysogenic decision upon infecting bacteria. During the lytic cycle, progeny phages are produced and the cell lyses, and in the lysogenic cycle, P1 DNA exists as a low-copy-number plasmid and replicates autonomously. Previous studies at the bulk level showed that P1 lysogenization was independent of multiplicity of infection (MOI; the number of phages infecting a cell), whereas lysogenization probability of the paradigmatic phage λ increases with MOI. However, the mechanism underlying the P1 behavior is unclear. In this work, using a fluorescent reporter system, we demonstrated this P1 MOI-independent lysogenic response at the single-cell level. We further observed that the activity of the major repressor of lytic functions (C1) is a determining factor for the final cell fate. Specifically, the repression activity of P1, which arises from a combination of C1, the anti-repressor Coi, and the corepressor Lxc, remains constant for different MOI, which results in the MOI-independent lysogenic response. Additionally, by increasing the distance between phages that infect a single cell, we were able to engineer a λ-like, MOI-dependent lysogenization upon P1 infection. This suggests that the large separation of coinfecting phages attenuates the effective communication between them, allowing them to make decisions independently of each other. Our work establishes a highly quantitative framework to describe P1 lysogeny establishment. This system plays an important role in disseminating antibiotic resistance by P1-like plasmids and provides an alternative to the lifestyle of phage λ.

## INTRODUCTION

**FIG 1:**
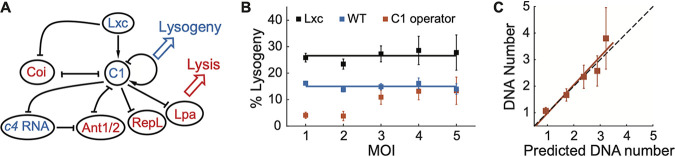
The probability of lysogeny is independent of the number of infecting phages at the single-cell level. (A) Schematic of the P1 lysis-lysogeny regulatory network. (B) Probability of lysogeny as a function of MOI. The probability of lysogeny remains constant over different MOI for wild-type (WT) cells at ∼14.53% (blue; cell sample sizes for MOI of 1 to 5 are 457, 277, 168, 62, and 68, respectively) and for Lxc-overexpressing cells at ∼26.58% (black; cell sample sizes for MOI of 1 to 5 are 295, 132, 55, 38, and 15, respectively). For cells with more C1 operator sequences (red; cell sample sizes for MOI of 1 to 5 are 147, 111, 44, 14, and 9, respectively), the probability of lysogeny increases with MOI at lower MOI and returns to the normal level at MOI of >3. Error bars denote counting errors. (C) The injected DNA copy number correlates with the prediction very well (see also Fig. S2C). Cell sample sizes for MOI of 1 to 5 are 83, 33, 17, 14, and 5, respectively. Filled squares, experimental data; red line, linear regression fit; black dashed line, diagonal indicating the perfect positive correlation. Error bars denote standard errors of the means.

Bacteriophages and phage-like plasmids play a prominent role in the dissemination of adaptive traits that allow stable colonization and persistence of multidrug resistance in pathogenic bacteria (1–12). A better understanding of their contribution to pathotype spread and adaptation is important. Phage P1 was discovered in a lysogenic strain of Escherichia coli named Li about 70 years ago (13). In terms of the ability to inject its DNA, P1 has the broadest known host range, including a variety of Gram-negative enteric bacteria, e.g., E. coli, Shigella dysenteriae (13), and Salmonella enterica serovar Typhimurium (14–17), and soil bacteria, including Pseudomonas aeruginosa (17, 18), Acetobacter suboxydans (17), and Myxococcus xanthus (19, 20). In the E. coli host, the infection cycle of P1 begins with adsorption to the cell surface by recognizing the terminal glucose moiety of the lipopolysaccharide (LPS) core region (21). The interaction of at least three of the six tail fibers with receptor molecules is assumed to be sufficient to stimulate the injection process (22). Injected viral DNAs can be protected from host type I restriction-modification (RM) system by P1 antirestriction proteins (23–25). Following DNA injection, the infected cell enters either the lytic cycle, where progeny phages are produced and the cell lyses, or the lysogenic cycle, in which the phage DNA exists as a low-copy-number plasmid and replicates autonomously. At the molecular level, the lysis-lysogeny decision is controlled by the interactions between phage components of the regulatory network (Fig. 1A). Briefly, the major repressor of P1, C1, is expressed immediately after phage infection and binds to 22 operator sequences widely dispersed over the P1 DNA to repress lytic gene expression. For example, C1 controls the expression of RepL, which is responsible for vegetative DNA replication, and Lpa, which activates the late promoter serving viral morphogenesis and lysis genes (26–28). Coi (*c one* inactivation) is an antirepressor that binds to C1 and inactivates its function (29–31), whereas the corepressor Lxc (lowers expression of *c1*) increases C1 binding affinity by forming a ternary complex with C1 and operator DNA and inhibits the ability of Coi to dissociate the operator-C1 complex (32–34). Moreover, the heterodimeric complex of Ant1 and Ant2 proteins also functions as an antirepressor, while the *trans*-acting *c4* RNA represses the synthesis of Ant proteins (Fig. 1A) (for a detailed review, see reference 35).

10.1128/mBio.01013-21.2 FIG S2 Monitoring of viral DNA injection during P1 infection using the *tetO*/TetR-FP DNA virtualization system. (A) Schematic of phage DNA labeling through TetR-mNeonGreen binding in E. coli strain LZ2001. (B) Representative images showing phage DNAs (green spots) labeled with TetR-mNeonGreen during the infection of gp23-mTurquoise2 phage P1LZ2504 (red spots). (C) The injected DNA copy number correlates with the predicted DNA copy number very well. Cell sample sizes for MOI of 1 to 4 are 144, 65, 16, and 12, respectively. Filled squares, experimental data; green line, linear regression fit; black dashed line, diagonal indicating the prefect positive correlation. Error bars denote standard errors of the means. See also Fig. 1C. Download FIG S2, TIF file, 2.2 MB. Copyright © 2021 Zhang et al. 2021 Zhang et al. https://creativecommons.org/licenses/by/4.0/ This content is distributed under the terms of the Creative Commons Attribution 4.0 International license.

Some studies suggest that the initial choice between lysis and lysogeny relies on the relative synthesis rate of the main competing players, C1 and Coi (28), but no quantitative evidence has been available to determine the relationship between this competition and the final lysis-lysogeny decision. In addition, the benefits of P1 acquiring different regulatory components besides the main competing players are not clear. Regrettably, studies of P1 regulatory circuitry halted prematurely with the death of their prime mover, Heinz Schuster (30, 31, 33–38). Furthermore, interest in P1 decreased, since it does not contain cargo genes of clinical interest (e.g., stress resistance and nutrient acquisition), and, being a temperate phage, P1 has been not considered for antibacterial therapeutic applications (phage therapy). However, recent advances in sequencing technologies have allowed the identification of numerous P1-like plasmids in bacteria and human pathogens (2, 4–6, 9, 11, 12), suggesting their abilities to serve as specific vehicles for the spread and the maintenance of virulence and antimicrobial resistance in both clinical and livestock production settings (1, 9). For example, pTZ20_1P, isolated from a porcine commensal E. coli strain and carrying multiple antibiotic resistance markers, was able to lysogenize a commensal E. coli strain with consequent transfer of resistance (9). Moreover, the sequencing data showed that genes contributing to lysogeny establishment were very similar to the genes of the regulatory network of P1 (9). Therefore, a thorough study of P1 life cycle and mechanism of its lysogeny establishment is important to understand the phage-driven antimicrobial resistance emergence and pathogen adaptation, which will benefit the development of novel therapeutic methods.

Studies of other temperate phages revealed that the lysis-lysogeny decision is highly dependent on the host availability in the environment or the virus-cell ratio. For phage λ, one of the best-studied paradigms for cell-fate decision-making, the probability of lysogeny increases with multiplicity of infection (MOI; the number of phages infecting a cell) and approaches 100% when the MOI is sufficiently high (39–41). Upon infection of the *Vibrio* phage 882, a quorum sensing factor expressed from the host cell is utilized to make the lysis-lysogeny decision (42, 43). Moreover, during infection, some *Bacillus* phages produce a 6-amino-acid peptide, designated arbitrium, that accumulates in the medium. In turn, high levels of arbitrium cause subsequent phage infections to be biased toward lysogeny (44, 45). These and other strategies are used by the phages to determine whether there are enough host cells for the infection by progeny phages, ensuring a high efficiency of genome propagation. In contrast, phage P1 has been reported to lack sensitivity to the virus-cell ratio. Previous studies by Bertani and Nice (46) and Rosner (47) at the bulk level showed constant probabilities of lysogeny regardless of MOI: 80 to 90% of S. dysenteriae cells were lysogenized at 20°C when infected by the P1 strain isolated originally from the lysogenic E. coli strain Li (13); ∼30% of the E. coli cells were lysogenized at 30°C by the thermoinducible P1 strain (P1CM*c1-*100) (47). The constant probability of lysogeny irrespective of host availability, together with P1’s capacity for autonomous prophage maintenance, utilizing systems such as type III RM (48–50), plasmid replication (51, 52) and toxin-antitoxin (53, 54), is a strategy used by P1 to propagate its genomic information as a plasmid. Furthermore, the ability to establish and be maintained as a plasmid can also be beneficial to the dissemination of the cargo genes carried by P1 or P1 phage-like elements.

In this work, we set out to examine the infection process of phage P1 at the levels of individual phage and cell with a spatiotemporal resolution sufficient to quantify the relevant subcellular parameters and to evaluate the contribution of each parameter to the observed cell-fate decisions. Using our system, we first investigated the mechanism of P1 MOI-independent lysogenization by linking the regulatory gene expression with cell fate by a simple genetic model. We also found that MOI dependency can be imposed by increasing the distances between the coinfecting phages. Taken together, our results provide a new decision-making model that expands our understanding of temperate phages. This, in turn, gives us insight into how the fitness of different phages results in distinctive decision-making behaviors and also allows us to evaluate their contributions to pathogenicity development.

## RESULTS

### The probability of lysogeny is independent of the number of infecting phages at the single-cell level.

In order to study P1 lysogenization at the single-cell level, we constructed a fluorescent reporter system to monitor individual decision-making events under the microscope, as described previously (55). Briefly, to record the number of phages infecting each E. coli cell, P1 virions were labeled by fusing fluorescent proteins to phage capsids. Moreover, during the time-lapse movies, the lytic pathway was followed through a reporter plasmid expressing mTurquoise2 from the PL23 promoter, which is dependent on the late gene activator, Lpa; the indicator for the lysogenic pathway was the expression of C1-mVenus fusion proteins followed by cell division (Fig. S1). Using this reporter system, we examined the effect of MOI on the cell fate. In agreement with previous bulk experiments using P1-carried chloramphenicol resistance as a readout (47), our single-cell measurements demonstrated that the probability of lysogeny is constant over different MOI (Fig. 1B).

10.1128/mBio.01013-21.1 FIG S1 Representative images from a time-lapse movie depict infection events using the fluorescent reporter system. Shown is an overlay of the phase-contrast, CFP (sum of multiple z-stacks for 0 min; single *z* stack at later time frames), and YFP channels. At 0 min, an E. coli cell on the left is infected by a single P1 phage and a cell on the right by two P1 phages (cyan spots). At 30 min, both cells show mVenus signal, indicating that viral DNAs are successfully injected and phage C1 protein is expressed. At 75 min, the cell on the left divides and grows normally, indicating entrance into the lysogenic pathway; whereas the cell on the right has gone into the lytic pathway, as indicated by the production of gp23-mTurquoise2 from the promoter LP23 on the lytic reporter plasmid. At 100 min, the lytic pathway has resulted in cell lysis (right) and the lysogenic cell (left) keeps dividing. Bar = 2 μm. Download FIG S1, TIF file, 1.3 MB. Copyright © 2021 Zhang et al. 2021 Zhang et al. https://creativecommons.org/licenses/by/4.0/ This content is distributed under the terms of the Creative Commons Attribution 4.0 International license.

Considering that some proteins encoded by P1, e.g., the superimmunity protein Sim, may act to prevent superinfecting phages from injecting their DNAs into the cytoplasm of infected cells (56, 57), we first tested if all the phages attached on the cell surface were able to inject their DNAs. To examine the number of injected DNAs, we utilized our established DNA visualization system by taking advantage of the specific binding of SeqA proteins to the methylated GATC sites (55, 58, 59). In particular, during phage infection, SeqA-mKate2 fusion proteins constitutively expressed in the methylation-deficient host cells (LZ1387, a Δ*dam* variant) bind to the methylated P1 DNAs but not the host DNA, allowing us to track the viral DNAs as fluorescent spots. Then, the injected-DNA copy number of each infected cell was calculated based on the total fluorescent intensity of all DNA spots in the cell at 0 min divided by the single-DNA intensity (see details in Materials and Methods). Meanwhile, as each phage particle was fluorescently labeled by mTurquoise2, we could count the phages that attached on the cell surface and predict the injected-DNA number based on failed and dark infection frequencies (see details in Materials and Methods). From our data, we observed a nearly perfect positive correlation between the actual injected-DNA number and the predicted DNA copy number (Fig. 1C and Fig. S2C). This indicated the success of multiple infection and the independence of the DNA injection process from different phages adsorbing to the same cell.

### A simple genetic model to elucidate MOI-independent decision-making.

**FIG 2:**
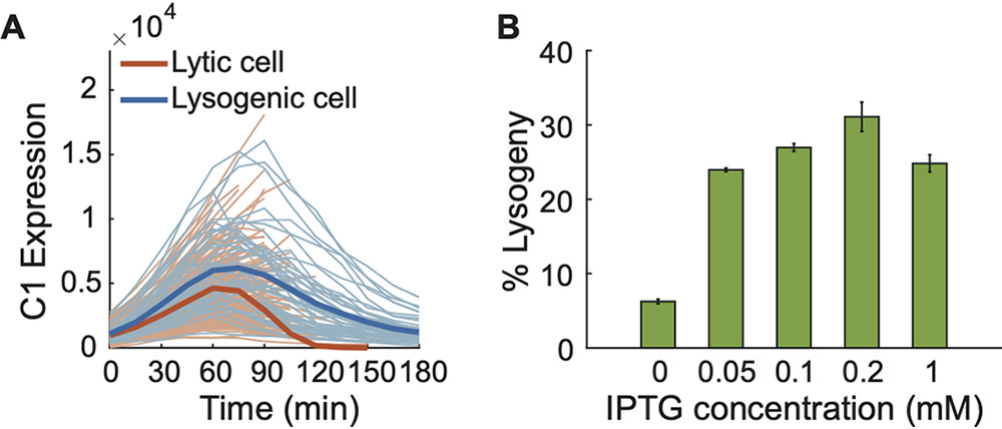
Influence of C1 activity on the probability of P1 lysogeny. (A) The average expression level of C1 (mVenus intensity) in lysogenic cells is higher than the level in lytic cells. Bold lines, mean intensities; light lines, C1 expression trajectories of each individual cell. Blue, lysogenic cells (*n* = 47); red, lytic cells (*n* = 165). (B) Probability of lysogeny upon the infection of E. coli cells with different expression levels of Lxc at the bulk level (MOI = 1). Lxc was induced with different concentrations of IPTG from the PLacO promoter on a plasmid. The error bars denote counting errors.

To understand the underlying mechanism of this MOI-independent cell lysogeny, we sought a minimal genetic model based upon the regulatory network of P1 (Fig. 1A). Given the central role of the major repressor, we hypothesized that C1 activity is a cell fate determining factor. Based on P1 regulatory circuitry, C1 activity can be affected by different parameters, e.g., the expression level of C1 compared with its inhibitor Coi, the level of the corepressor Lxc, and the number of intracellular C1 binding sites (22 sites per P1 DNA). We first compared C1 expression level in lytic and lysogenic cells by tracking C1-mVenus intensity using the decision-making reporter system (55). As expected, cells entering the lysogenic pathway showed higher C1 signals on average than those entering the lytic pathway, though it was not always true for every single-cell trajectory (Fig. 2A). Next, we tested whether the decision-making outcome could be manipulated by varying the intracellular C1 activity. To enhance C1 activity, we overexpressed the corepressor Lxc from plasmids in host cells. Indeed, host cells with higher Lxc expression levels exhibit higher probabilities of lysogeny at both the bulk level (Fig. 2B) and the single-cell level (Fig. 1B). Interestingly, the probability of lysogeny was still independent of MOI (Fig. 1B). On the other hand, to reduce the C1 activity, we introduced more C1 operator DNA sequences from plasmids (∼15 sites per cell) and found a reduction of the lysogeny probability at low MOI (1 and 2) (Fig. 1B), although not at higher MOI, where changes of the number of C1 binding sites were relatively low and hence showed less effect on C1 function. In summary, these data suggest that C1 activity is a determining factor of P1 decision-making.

**FIG 3:**
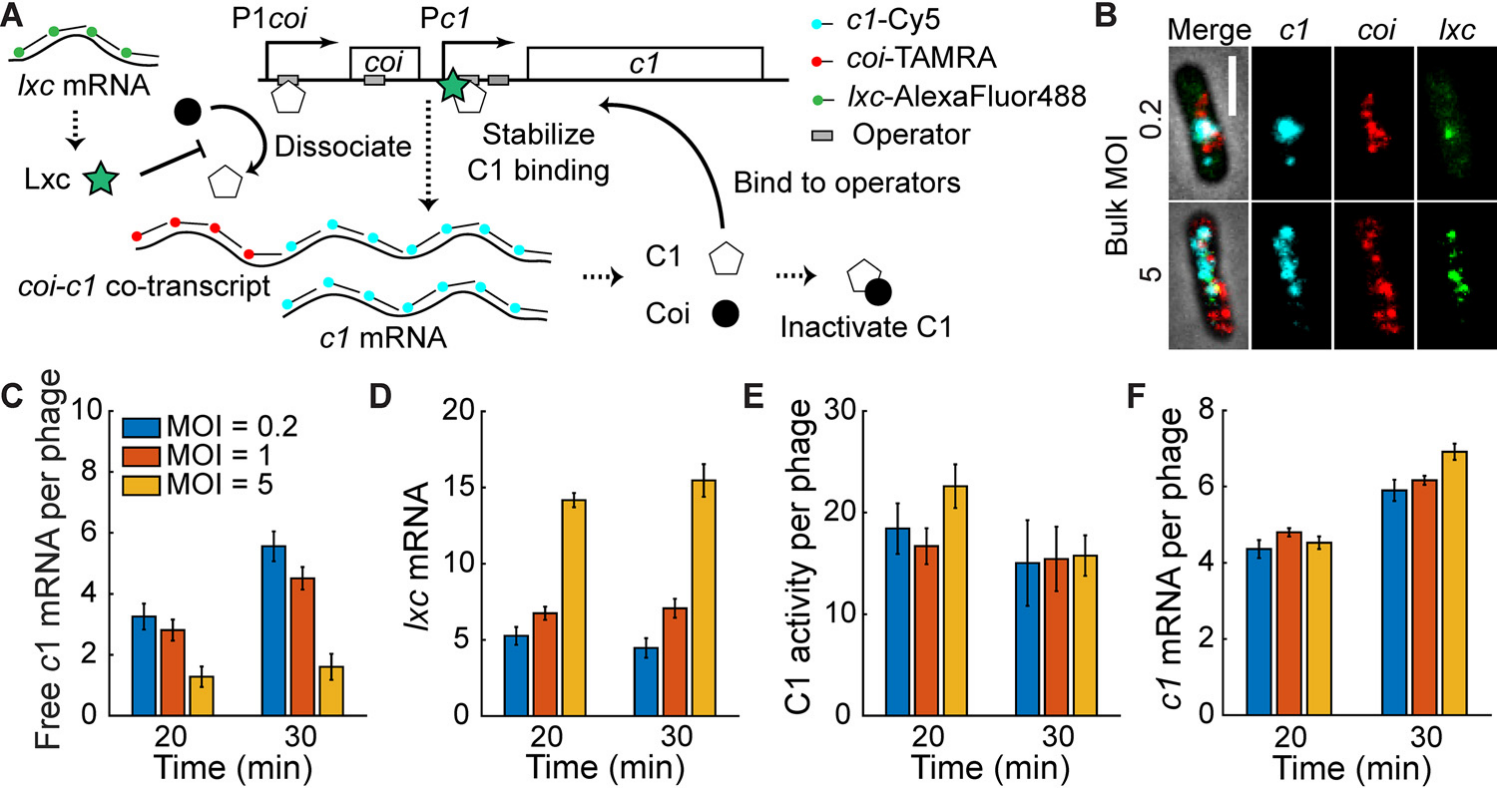
The interaction of C1, Coi, and Lxc leads to the constant C1 activity of each phage despite different MOI. (A) Schematic of the *coi-c1* operon and the smFISH method. C1 is expressed from two promoters (P1*coi* and P*c1*), while Coi is expressed only from P1*coi*. C1 is able to inhibit both P1*coi* and P*c1* by binding to the adjacent operator sequences. Coi forms a 1:1 complex with C1 to inactivate C1 function. Lxc promotes C1 binding affinity to its operators and inhibits the dissociated function of Coi. (B) Representative images showing mRNA expression at 30 min after infection at bulk MOI of 0.2 (top) and 5 (bottom). Cyan, *c1*; red, *coi*; green, *lxc*. Bar = 2 μm. (C) Free *c1* mRNA per phage decreases with MOI. See also Fig. S5. (D) The level of *lxc* mRNA increases with MOI. (E) The C1 activity of each infecting phage, i.e., free *c1* mRNA times *lxc* mRNA per phage, is similar at different MOI. (F) In Lxc-overexpressed host cells, the lysis-lysogeny decision is determined by the level of C1 per phage. smFISH experiments show that the level of *c1* mRNA per phage is similar at different MOI. In all plots, error bars denote standard errors of the means.

We then hypothesized that the C1 activity of each infecting phage was constant in cells with different MOI, which led to the MOI-independent lysogenization. Initially, we defined the total C1 activity in an infected cell as the amount of C1 that is not bound by Coi (or free C1) based on their antagonistic functions and assumed that the amount of free C1 per infecting phage was a constant. To test this, we compared mRNA levels of *c1* and *coi* simultaneously during P1 infection at different bulk MOI (0.2, 1, and 5), measured by single-molecule fluorescence *in situ* hybridization (smFISH) (60). DNA FISH and quantitative PCR (qPCR) data (Fig. S3) suggested that the viral DNA replication began around 30 min after infection, so we focused on mRNA levels at 20 min and 30 min. Fluorescent probes were designed to target *c1* or *coi* mRNA so that we could quantify them separately (Fig. 3A and B). According to the Poisson distribution (55), at bulk MOI of 0.2, 1, and 5, the estimated proportions of infected cells out of total cells were about 18%, 63%, and 99%, respectively. From the smFISH experiments, the percentage of infected cells (cells showing *c1* or *coi* mRNA signal) were consistent with our predictions (Fig. S4). However, the difference between *c1* and *coi* mRNA per phage, i.e., free *c1* mRNA per phage, decreased with MOI instead of remaining constant (Fig. 3C), where the mRNA levels of both *c1* and *coi* per phage decreased with MOI (Fig. S5). This suggested that, in order to achieve MOI-independent lysogenization, there would be other components that work together to diminish the effect of MOI.

10.1128/mBio.01013-21.3 FIG S3 P1 DNA replication during infection. (A) Plot of DNA copy number over time tested by DNA FISH at a bulk MOI of 0.2. Error bars denote standard errors of the means. (B) Plot of DNA copy number over time tested by qPCR at a bulk MOI of 1. Both data indicate that the replication of P1 DNA starts around 30 min after phage infection. Download FIG S3, TIF file, 1.6 MB. Copyright © 2021 Zhang et al. 2021 Zhang et al. https://creativecommons.org/licenses/by/4.0/ This content is distributed under the terms of the Creative Commons Attribution 4.0 International license.

10.1128/mBio.01013-21.4 FIG S4 Percentage of infected cells over total cells tested by smFISH. Red squares show the experimental data of the percentage of infected cells over time during P1LZ1856 infection. Black dashed lines show the predicted infection rates of 18%, 63%, and 99% at bulk MOI of 0.2, 1, and 5, respectively, calculated based on Poisson distribution. The error bars denote counting errors. Download FIG S4, TIF file, 1.3 MB. Copyright © 2021 Zhang et al. 2021 Zhang et al. https://creativecommons.org/licenses/by/4.0/ This content is distributed under the terms of the Creative Commons Attribution 4.0 International license.

10.1128/mBio.01013-21.5 FIG S5 mRNA level of *c1* and *coi* during the infection P1LZ1856 of WT host strain MG1655. (A) The level of *c1* mRNA per phage decreases with MOI. (B) The level of *coi* mRNA per phage decreases with MOI. See also Fig. 3C. Download FIG S5, TIF file, 0.9 MB. Copyright © 2021 Zhang et al. 2021 Zhang et al. https://creativecommons.org/licenses/by/4.0/ This content is distributed under the terms of the Creative Commons Attribution 4.0 International license.

Based on the aforementioned regulated function of Lxc, we decided to define C1 activity considering the expression level of both free C1 and Lxc. Since none of the promoters that drive the transcription of gene *lxc* is under the control of C1 or other factors, we hypothesized that Lxc is constitutively expressed upon infection and the Lxc level is positively correlated with MOI. As shown in Fig. 3D, *lxc* mRNA number increased with MOI from smFISH experiments. Together with the observation that free *c1* mRNA per phage decreased with MOI (Fig. 3C), our simple model became one in which the C1 activity of each infecting phage, defined as the expression level of free C1 times the expression level of Lxc per phage, remained constant over MOI. To test this, we quantified the mRNA expression of *c1*, *coi*, and *lxc* in the same cell during phage infection using smFISH. Indeed, our results showed that the C1 activities per phage were similar at different bulk MOI (Fig. 3E). These data support our model for MOI-independent lysogeny and indicated the importance of Lxc for maintaining a set frequency of lysogeny during P1 infection.

Next, we assumed that the proportional expression level of Lxc over MOI was the key to maintaining the probability of cell lysogeny. To demonstrate this, we overexpressed Lxc; hence, its level became a constant in all infection events, which should have resulted in MOI-dependent lysogeny. However, based on the data from our single-cell infection assay, probabilities of P1 lysogeny during the infection of the Lxc-overexpressed hosts were still independent of MOI (Fig. 2B). The mechanism behind it would be that, with this saturated Lxc function on C1 activity, the function of Coi in inactivating C1 was abolished. In this scenario, cell fate should be simply determined by the level of C1 per phage. From the smFISH experiment upon the infection of Lxc-overexpressing hosts, we indeed observed a constant level of *c1* mRNA per phage at different MOI (Fig. 3F), which led to the MOI independency. In summary, C1 activity regulating each phage depends on the expression level and the interaction between the gene products of *c1*, *coi*, and *lxc* and is a constant during infection with different numbers of P1 virions, which results in an MOI-independent lysogenic response.

### Imposing MOI dependency by increasing the distance between infecting phages.

**FIG 4:**
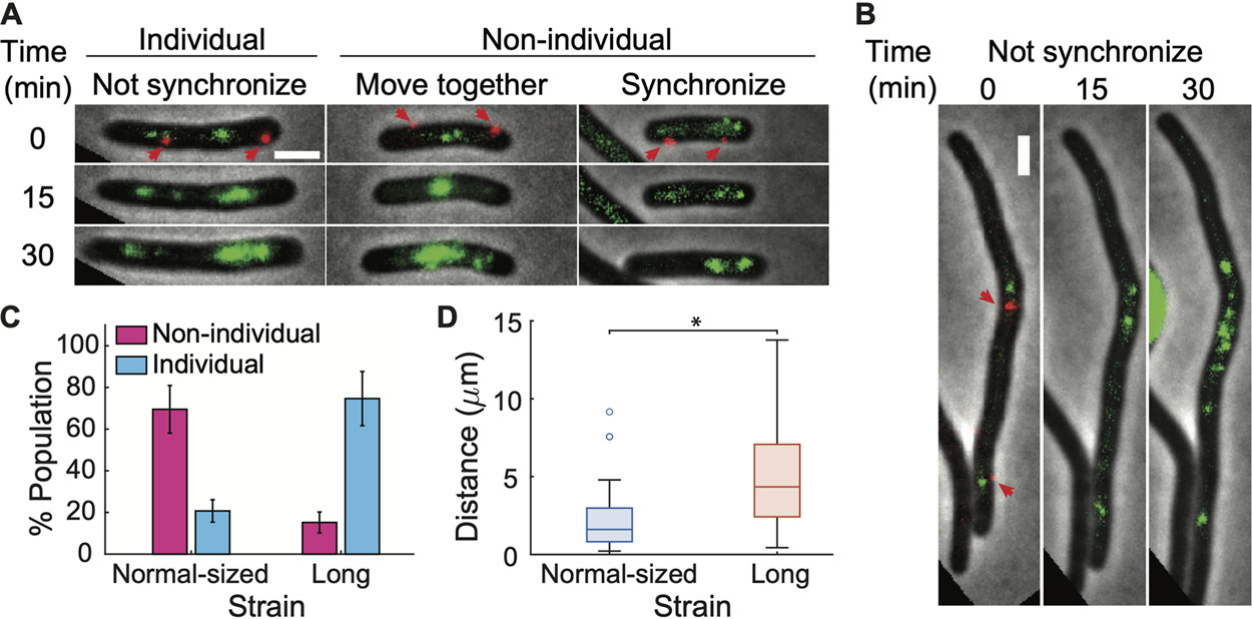
P1 virions infecting the same host cell make an ensemble lysis-lysogeny decision. (A) Representative images showing phage DNA behaviors in normal-sized E. coli cells at an MOI of 2 (green signals represent TetR-mNeonGreen-bound phage DNAs). (Left) One of the phage DNAs replicated earlier or faster than the other, resulting in an unsynchronized pattern. (Middle) Phage DNAs physically moved together. (Right) Phage DNAs located at different cell areas showed synchronized replication patterns. The MOI was determined by the number of mTurquoise2 fluorescent phages (red dots indicated by red arrows) attached on the cell surface at 0 min. (B) Representative images showing unsynchronized phage DNA behaviors in long cells with λKil expression at an MOI of 2. Bar = 2 μm. (C) Bar plot showing more nonindividual DNA behaviors in normal-sized cells (*n* = 82) versus more individual phage DNA behaviors in long cells (*n* = 79). The error bars denote counting errors. (D) Box plot of the distance between coinfecting phages on the surface of long cells (red, *n* = 121) is much larger than that in normal-sized cells (blue, *n* = 113) at an MOI of 2. *, *P* < 0.001 as determined by Student's *t* test. See also Fig. S6.

Recent studies showed that phage λ exhibits individual lysis-lysogeny decision-making by each infecting phage, as evidenced by phage DNA integration into the host chromosome (lysogeny) followed by cell lysis as well as the coexpression of capsid decoration proteins (gpD) from different infecting phages, indicative of lytic pathways, coexpression of CI from different infecting phages, indicative of lysogenic pathways, and coexpression of both gpD and CI, indicative of both lysis and lysogeny in single cells (59, 61, 62). The decisions by all λ phages infecting a single cell are then integrated into the final cell fate (41, 59). In the meantime, the probability of lysogeny of phage λ increases with MOI (39–41). We speculate that the MOI-dependent lysogenization of λ is correlated with the individual phage lysis-lysogeny decision. Here, the MOI independency of P1 lysogenization may then imply that the infecting phages make an ensemble decision. To explore this, we examined P1 DNA interactions inside the cell using a *tetO/*TetR-FP DNA visualization system (Fig. S2A and B) (61, 63). Basically, we replaced *darB* (23, 64), a gene nonessential for P1 decision-making, with a 120×*tetO* array in the phage DNA. Upon infection of the host cell harboring a plasmid constitutively expressing TetR-mNeonGreen fusion proteins, all injected and replicated phage DNAs are bound and labeled at *tetO* sites by fluorescent TetR-mNeonGreen proteins, forming foci or clusters (Fig. 4A and B). During the infection process, some interesting DNA behaviors were detected (Fig. 4A): (i) one of the phage DNAs replicated earlier or faster than the other, resulting in an unsynchronized pattern; (ii) phage DNAs physically moved together; and (iii) viral DNAs located at different cell areas showed synchronized replication patterns. Asymmetric levels of DNA replication might be due to the uneven distribution of intracellular machineries, suggesting that phages made decisions individually, defined as individual DNA behavior. In contrast, DNAs that move together or show synchronized replication patterns would probably indicate that the infecting phages shared regulatory products to regulate each other and made decisions as one, i.e., nonindividual behavior. A majority of the cells (∼70%) exhibited the nonindividual behavior (Fig. 4C and Fig. S6A).

10.1128/mBio.01013-21.6 FIG S6 Interaction between coinfecting phages with different distances. (A and C) Probability of cells with different DNA behaviors in normal-sized and long cells (see also Fig. 4C). Error bars denote counting errors. (B and D) Box plots exhibit the distances between phages with varied interaction behavior in normal-sized and long cells at an MOI of 2 (see also Fig. 4D). Others indicate the cell with only one phage DNA spot at 0 min, probably due to failed infection. Download FIG S6, TIF file, 2.2 MB. Copyright © 2021 Zhang et al. 2021 Zhang et al. https://creativecommons.org/licenses/by/4.0/ This content is distributed under the terms of the Creative Commons Attribution 4.0 International license.

**FIG 5:**
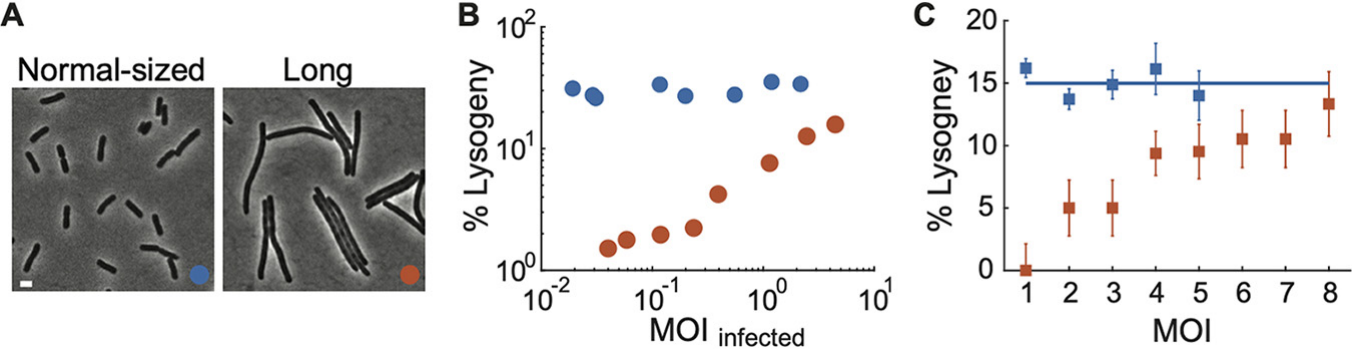
MOI-dependent lysogenization was increased by increasing the distance between coinfecting phages. (A) Representative images of normal-sized cells and long cells. Bar = 2 μm. (B) The probability of lysogeny increases with MOI for long cells (red) and remains constant for normal-sized cells (blue), tested at the bulk level (see also Fig. S7). Red circles, λKil was induced using 0.05% arabinose from plasmid pBAD to generate long cells. Blue circles, no arabinose treatment. (C) Comparison of the probability of lysogeny increasing with MOI upon infection of normal-sized cells and long cells at the single-cell level. Orange, long cells (cell sample sizes for MOI of 1 to 8 are 22, 20, 20, 32, 21, 19, 19 and 15, respectively); blue, normal-sized cells (see also Fig. 1B). Error bars denote counting errors.

The nonindividual behavior could be due to phages being physically close to each other and their gene products for lysis-lysogeny decision being easily shared among them. Therefore, a larger distance between infecting phages would inhibit the efficient *trans* regulation and thereby lead to individual decision-making. To test whether this is the case, we increased the distances of phages by infecting a much longer cell (Fig. 5A and Fig. S7A and B). Increased cell length was achieved by expressing the Kil protein of phage λ from a plasmid; Kil inhibits E. coli cell division, causing cells to grow into long filaments (65, 66). For simplicity, we analyzed the infected cells only at an MOI of 2. Using the *tetO/*TetR-mNeonGreen DNA visualization system, we observed more unsynchronized DNA replications during the infection of long filamentous cells (Fig. 4B), which gave rise to a higher level of the individuality of coinfecting phages (Fig. 4C and D; Fig. S6C and D). Next, we tested P1 lysogenic response in long cells. In both bulk and single-cell experiments, the probability of cells undergoing lysogeny increased with MOI instead of being a constant value (Fig. 5B and C; Fig. S7C and D). In summary, these data indicate that infecting P1 phages are more likely to make the ensemble decision and tend toward MOI-independent lysogenization when they can efficiently regulate each other in the scenario of normal-sized cells; on the other hand, phages can make individual decisions and tend toward an MOI-dependent lysogenic response when the distance between them is large enough to diminish *trans* regulation.

10.1128/mBio.01013-21.7 FIG S7 P1 shows MOI-dependent lysogenic response in long cells tested at the bulk level. (A) Representative images for the comparison of cell lengths between normal-sized (blue) and long cells (red, 0.05% arabinose for λKil induction; green, 0.4% arabinose for λKil induction). Bar = 2 μm. (B) Quantification of cell lengths from panel A. The mean lengths are 4.63 μm (*n* = 231), 13.69 μm (*n* = 110), and ≥26.36 μm (*n* = 37; some long cells could not be measured since they were longer than the imaging field of the microscope) for normal-sized and long cells induced with 0.05% and 0.4% arabinose, respectively. *, *P* < 0.001 as determined by Student’s t test. (C) Probability of lysogeny calculated based on the number of total cells. P1 shows different lysogenic responses in cells with different lengths as function of bulk MOI (total phage/total cell). Data are shifted with the maximum lysogeny of 100% in order to compare to the Poisson distribution. In normal-sized hosts (blue spots), the probability of P1 lysogeny follows the theoretical prediction of a Poisson distribution of *n* ≥ 1 (solid black line). In long cells induced cells with 0.4% arabinose (green spots), the probability of P1 lysogeny follows the theoretical prediction of a Poisson distribution of *n* ≥ 2 (dashed black line), which is similar to the probability of lysogeny upon the infection of phage λ of normal-sized host cells (grey diamond) (58). The probability of P1 lysogeny in long cells induced with 0.05% arabinose (red spots) is located between the Poisson distributions of *n* ≥ 1 and *n* ≥ 2. (D) The probability of lysogeny is calculated based on the number of infected cells. In normal-sized host cells, it shows a constant lysogenic response over MOI_infected_, which is (total phage − nonadsorbed phage)/total cell. However, the probability of lysogenization in long cells is lower than in normal-sized cells and increases with MOI_infected_ (see also Fig. 5B and C). (C and D) Open and filled circles are data from two independent experiments. Download FIG S7, TIF file, 1.6 MB. Copyright © 2021 Zhang et al. 2021 Zhang et al. https://creativecommons.org/licenses/by/4.0/ This content is distributed under the terms of the Creative Commons Attribution 4.0 International license.

## DISCUSSION

**FIG 6:**
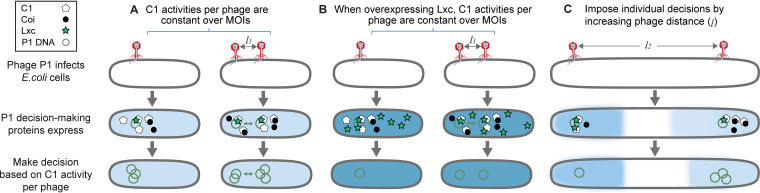
Model of the P1 lysis-lysogeny decision-making mechanism. (A) The lysis-lysogeny decision-making of P1 is independent of MOI. All infecting phages make a group decision inside the host cell due to *trans* regulation (green double arrow). The constancy of C1 activity of each phage, depending on the expression levels of C1, Coi and Lxc, results in a robust lysogenic response against the number of phages infecting an individual cell. (B) The lysis-lysogeny decision-making of P1 is independent of MOI in the Lxc-overexpressing host. When Lxc is overexpressed, Coi function in inactivating C1 is abolished. The C1 level of each infecting phage becomes the determining factor for the lysis-lysogeny decisions, which is a constant value over different MOI, resulting in MOI independency. The C1 binding affinity or C1 activity per phage increases and leads to higher probabilities of lysogeny. (C) A different lysogenic pattern is imposed by increasing phage distances infecting the same host cell. It suggests individuality of the choice between lysis and lysogeny of individual phages that are not able to share regulatory components effectively. Blue intensities indicate C1 activities of each infecting phage, and darker color evinces higher activity, which is regulated by the expression levels and interactions between C1 (white pentagons), Coi (black circles), and Lxc (green stars).

In this work, we investigated the underlying mechanism of how P1 enters its lysogenic state, in terms of its gene regulatory circuitry, which is conserved in P1-like plasmids (9). By taking advantage of single-cell techniques, we first demonstrated the MOI-independent lysogenic response at the single-cell level (Fig. 1B) and then proposed its mechanism with a simple model: The C1 activity of each infecting phage, depending on the relative expression level and interactions between C1, Coi, and Lxc, was regulated to be a constant value over different MOI, resulting in the similar probabilities of lysogeny (Fig. 6A). When Lxc was overexpressed, Coi function in inactivating C1 was abolished and the C1 level of each infecting phage became the determining factor for the lysis-lysogeny decisions, which was a constant value over different MOI, resulting in MOI independency (Fig. 6B). Given that Lxc has no homologs in phage λ, the function of Lxc in maintaining the probability of lysogeny suggests that phage P1 uses a totally different gene regulation at the molecular level to guide its lysis-lysogeny decision. Furthermore, compared with the individuality of phage λ for decision-making (41, 59, 61, 62), the independence of MOI for the phage P1 lysogenic response strongly signals that the infecting phages make a group decision.

To test this hypothesis, we examined the interaction of injected P1 DNAs in normal-sized E. coli cells with an MOI of 2 and observed more nonindividual DNA behaviors (∼70%) than the individual ones. Among these, the cases where the unsynchronized replication of P1 DNAs can be seen as phage individuality; i.e., coinfecting phages make different decisions due to the uneven intracellular environment. For example, the bacterial nucleoid is located in the center of the cytoplasm, leaving the polar regions largely free of DNA, while certain functional proteins, such as the osmosensory transporter ProP (67, 68), and ribosomes (68) are enriched at the cell poles. After the individual decision-making of each P1, the decisions by all phages probably integrate into the final cell outcome as lysis or lysogeny, similar to what occurs with phage λ. On the other hand, the majority of P1 DNAs exhibited nonindividual behaviors as they moved together or showed synchronized replication patterns. In addition, the proportion of nonindividuality was assumed to be even higher in the cells with higher MOI, given the increased viral concentration, which results in an overall MOI-independent cell outcome. Furthermore, we found that a large separation among P1 virions infecting the same cell prompted the individual decision-making and altered the lysogenic response to be dependent on MOI (Fig. 6C). This result highlights the important role of the spatial organization in the process of cell fate determination in a single-cell environment.

The striking differences between phage P1 and phage λ may suggest distinct strategies developed by these two phages for efficient genome propagation, which is essential for all organisms to survive in the fluctuating environment. Phage λ can infect only a subclass of E. coli strains and may therefore be considered a specialist, implying the limited availability of host cells under natural conditions. Additionally, phage λ uses the outer membrane protein LamB as its receptor, which shows helical or ring-like patterns on cell surfaces (69), contributing to the preferential adsorption to cellular poles (41, 70). The use of polar adsorption augments the infection specificity at the cost of a higher rate of failed infection (41, 55). Thus, the ability to sense the amount of host cells for the infection of progeny phages is important: the injected phage DNA is replicated and packaged into new progeny viral particles under conditions where host cells are not limiting, whereas viral DNA stays dormant in the host when not enough host is available. Moreover, the MOI dependency of λ has been found to rely on the individuality of each infecting phage; coinfecting phages make individual lysis-lysogeny decisions, and the host cell integrates these decisions to result in lysis, lysogeny, or lyso-lysis (71).

In contrast, phage P1 can be regarded as a generalist with the ability to inject its DNA into a very broad spectrum of Gram-negative hosts. It recognizes evenly distributed LPS molecule as its receptor, leading to the nonspecific adsorption to the cell surface (55). The broad host range and the high probability of successful DNA injection (55) reduce the need to sense the availability of host bacteria. It may also reflect a phage’s real-world experience of having to deal with diverse cytoplasmic environments where the lytic pathway may be problematic and less attractive in terms of fitness, as for the need for different strategies to replicate a large number of viral DNAs, while the establishment of a stable lysogen becomes preferred. Thus, P1 makes the lysis-lysogeny decision regardless of MOI and in general with a bias toward lysogeny. Furthermore, during lysogenization, P1 is maintained as a self-replicating plasmid (51, 52). First, the plasmid format gives P1 prophage more freedom from the host cell’s control, in processes such as DNA replication, where P1 uses its own origin and replication system (28), and protein synthesis, considering the effects of the chromosome position on the gene expression profile in bacteria (72). Second, given the aforementioned capacities for plasmid maintenance (48–54) and the separate distribution of genes with related functions on the viral genome (28), P1 may have evolved directly from an intracellular plasmid by acquiring phage-related elements. These suggest a distinct evolutionary lineage of P1 with other temperate phages. In sum, the formation of stable plasmids is probably the priority for phage P1 during infection, and a robust lysogenic response over host availability helps ensure this process. Essentially, the autonomy and constant lysogenization make P1 an ideal backbone to carry and spread cargo genes during virulence dissemination. On the other hand, the P1 phage particle is more likely to be used in lateral gene transfer between bacterial strains, which is less essential for P1 propagation.

## MATERIALS AND METHODS

### Media.

LB broth contained (per liter of distilled H_2_O) 10 g Bacto tryptone, 5 g Bacto yeast extract, and 5 g NaCl and was adjusted to a pH of about 7.5 with NaOH. Derivative media were supplemented with the following: LBM, 10 mM MgSO_4_, used for generating crude lysates and in agarose slabs for single-cell microscopy; LB plates, 1.5% agar; LBCM plates, 1.5% Bacto agar and 12.5 μg ml^−1^ chloramphenicol. SM buffer (50 mM Tris-HCl [pH 7.5], 100 mM NaCl, 8 mM MgSO_4_) was used as a phage storage buffer. NZYM (N-Z amine-NaCl-yeast extract-MgSO_4_) was used for phage titration, supplemented with 1.5% or 0.7% Bacto agar for plates and top agar, respectively. Neidhardt EZ rich defined medium (73) was used for DNA visualization experiments due to its low background and fluorescence. It contained (per liter of distilled H_2_O) 1× MOPS (morpholinepropanesulfonic acid) mixture (Teknova no. M2101), 1.32 mM K_2_HPO_4_ (Teknova no. M2102), 1× ACGU solution (Teknova no. M2103), and 1× supplement EZ (Teknova no. M2104). Carbon sources were added when the cell culture was started.

### Bacterial strains, phages, and plasmids.

All bacterial strains, plasmids, and phages are described in Table S1.

10.1128/mBio.01013-21.8 TABLE S1 Bacterial strains, phages and plasmids. Download Table S1, DOCX file, 0.02 MB. Copyright © 2021 Zhang et al. 2021 Zhang et al. https://creativecommons.org/licenses/by/4.0/ This content is distributed under the terms of the Creative Commons Attribution 4.0 International license.

**(i) Construction of Lxc expression plasmids (pLZ1931 and pLZ1933).** We constructed the plasmids containing the P1 *lxc* gene under the control of the IPTG (isopropyl-β-d-thiogalactopyranoside)-inducible promoter PLlacO-1. We inserted *lxc* (primers 1 and 2) into pZE12luc with NcoI and SphI (pLZ1931) and then transferred the whole fragment from PLlacO-1 to *mTurquoise2* (primers 3 and 4) into pACYC177 at the BamHI and PstI sites to maintain kanamycin resistance (pLZ1933). In order to achieve uniform IPTG induction, constructed plasmids were transformed into the MG1655 *lacI*^q^ Δ*lacY* strain for Lxc expression.

**(ii) Construction of the C1 operator plasmid (pLZ1950).** There are 22 C1 binding operators dispersed on the P1 genome. The 17-bp operator consensus sequence is asymmetric and is reported in reference 28 as ATTGCTCTAATAAATTT. One copy of the consensus operator sequence was designed in primer 5 and amplified with primer 6 from pACYC177. PCR fragments were ligated into pACYC177 between BamHI and PstI. We transformed the plasmid into MG1655 or other strains acting as the host cell during single-cell infection assays.

**(iii) Construction of *tetO* phage.** The *tetO* recombination plasmid (pLZ1981) was constructed to replace part of the *darB* region of P1KL1856. Specifically, a 120×*tetO*-Kan^r^ array digested from pLAU39 (63) (EcoRI and SalI), a homology region (H1) inside *darB* (primers 7 and 8), and a kanamycin resistance gene (primers 9 and 10) with its promoter were ligated into a pUC19 backbone using Gibson assembly. Another homologous region (H2) inside *darB* (primers 11 and 12) was then ligated into the assembled plasmid with SalI and HindIII. Next, we used the λ*red* recombination method (74) to recombine the *H1-*Kan^r^-120×*tetO-H2* sequence in the P1 genomic DNA. This DNA sequence was digested from pLZ1981 with NdeI and HindIII and transformed into LZ1856 lysogen with pKD46 under electroporation. Each electroporation reaction mixture contained 100 ng purified DNA and 100 μl competent cells in a 0.2-cm cuvette and was transferred in a Bio-Rad MicroPulser electroporator. Cells were recovered in 1 ml SOC (2% vegetable peptone, 0.5% yeast extract, 10 mM NaCl, 2.5 mM KCl, 10 mM MgCl_2_, 10 mM MgSO_4_, 20 mM glucose) solution for 1 to 2 h at 30°C and plated on LB plates containing Cm (12.5 μg ml^−1^) and Kan (30 μg ml^−1^) at 30°C overnight. The genomic construct was then verified by sequencing.

### Bacteriophage assays.

**(i) Production of phage lysates.** Phage lysates were produced by thermal induction of phage lysogens. Briefly, a single colony of the desired lysogen was grown in 1 ml LB with appropriate antibiotics overnight at 30°C. The overnight culture was diluted 100-fold in LBM and grown at 30°C until an optical density at 600 nm (OD_600_) of ∼0.5 was reached. P1 or a P1 mutant was thermally induced by shifting the culture to 42°C in the shaking water bath (180 rpm) for 30 min. Then, the culture was shaken at 37°C and 180 rpm for 1 h until an OD_600_ of ∼0.1 was reached. We collected the lysate in a centrifuge tube and mixed it with chloroform (2%) for about 15 min at room temperature (RT) for thorough lysis. The lysate was centrifuged at 10,000 × *g* for 10 min at 4°C and sterilized by the passage of the supernatant through a 0.2-μm filter (VWR International catalog no. 28145-477). The gp23-mTurquoise2 phages were obtained by inducing the lysogen (e.g., LZ1914 and LZ2504). During lysogen growth, IPTG was added to a final concentration of 0.075 mM when the OD_600_ approached 0.1, to provide the proper amount of gp23-mTurquoise2 for mosaic phage assembly.

**(ii) Phage titration assay.** An overnight culture of indicator strain MG1655 was diluted 100-fold in LBM and grown at 37°C to an OD_600_ of ∼0.4. Next, we kept the cell culture on ice and added 5 mM CaCl_2_. The phage stock was diluted in SM buffer to an estimated 10^3^ to 10^4^ PFU ml^−1^. We incubated 100 μl of the diluted phages with 300 μl of host solution for 20 min at RT. The phage-cell mixture was then added into 4 ml of 50°C molten NZYM top agar and plated on predried NZYM agar plates. The plates were allowed to set for 10 min at RT and incubated overnight at 42°C. The titer was determined as the ratio of plaques to the dilution factor.

**(iii) Phage purification by ultracentrifugation.** The crude lysate, obtained from the thermal induction in 500 ml LBM as described above, was centrifuged in a Sorvall GSA rotor at 10,000 rpm for 20 min at 4°C. The supernatant was transferred into a new bottle. After incubation with 1 μg ml^−1^ DNase and 1 μg ml^−1^ RNase for 1 to 2 h at RT, the lysate was concentrated by overnight centrifugation (∼16 h) in the Sorvall GSA rotor at 10,000 rpm, 4°C. We soaked the resulting phage pellet in 2 ml cold SM buffer and extracted the phage solution after a 48-h incubation. Phages were then purified by equilibrium centrifugation in 1.40 to 1.45 g ml^−1^ cesium chloride using the Beckman 70.1Ti rotor at 45,000 rpm for 24 to 26 h at 4°C. The phage band was extracted with 18-gauge needles and dialyzed against SM buffer in Slide-A-Lyzer 3,500-molecular-weight-cutoff (MWCO) dialysis cassettes (Thermo Scientific).

**(iv) Bulk lysogenization probability assay.** We measured the probability of lysogeny as a function of the MOI. An overnight culture of the host strain was grown in LB at 37°C. Cells were diluted 1,000-fold into LBM, grown at 37°C to an OD_600_ of ∼0.4, and chilled, and CaCl_2_ was added to a final concentration of 5 mM. Then, 100 μl of the cells was added to 100 μl of phage solutions with different concentrations. After a 30-min incubation at 30°C, we transferred 20 μl of the mixture into 980 μl ice-cold LB or SM buffer to stop the adsorption process. Aliquots in LB medium were plated on LBCM plates and incubated overnight at 30°C. Lysogen concentrations were determined by counting the number of Cm^r^ colonies. To examine the concentration of nonadsorbed phages, we centrifuged the aliquots in SM buffer and titrated the supernatant on MG1655. Total phage and bacterium concentrations were measured using plate assays as well. Bulk MOI was determined as total phages/total cells. The probability of lysogeny of all cells or of the infected cells (determined from the bulk MOI using the Poisson distribution with a cumulative probability of ≥1) was plotted as a function of the MOI on a log-log scale. MOI_infected_ was calculated as (total phages − nonadsorbed phage)/total cells. λKil expression in strains containing pBAD24-λ*kil* was induced with ʟ-arabinose when the OD_600_ approached 0.08. Lxc expression in strain LZ1931 was induced with IPTG when the OD_600_ approached 0.1.

### Single-cell infection assay.

**(i) Decision-making examination.** The protocol for single-cell infection assays was adapted from previous work (55). An overnight culture of LZ1915 was diluted 100-fold in LBM and grown to an OD_600_ of ∼0.4; then, CaCl_2_ was added to 5 mM. For infections in Lxc-overexpressing cells, IPTG was added at a final concentration of 0.2 mM to the cell culture (LZ1937) when the OD_600_ approached 0.1. Purified P1LZ1914 phages were mixed with the cells to reach an MOI of 0.1 to 7, followed by incubation for 30 min at 30°C to trigger both phage adsorption and viral DNA injection. One microliter of the sample was placed on a 1-mm 1.5% LB agarose pad resting on a small coverslip (18 by 18 mm; Fisher Scientific). After 1 to 2 min, a large coverslip (24 by 50 mm; Fisher Scientific) was gently overlaid, and the sample was imaged under the microscope at 30°C.

To localize all phages surrounding the cells, a series of 7 *z*-axis images with a spacing of 300 nm were taken through the cyan fluorescent protein (CFP) channel using a 500-ms exposure for each. Cells were imaged at multiple stage positions (typically 16) in each experiment. During the time-lapse movie, the sample was imaged in phase-contrast (100 ms exposure for cell recognition), yellow fluorescent protein (YFP) (300-ms exposure for phage C1-mVenus expression), and CFP (100-ms exposure for lytic reporter signal) channels at intervals of 5 min until cell fate was visible (3 h in total).

**(ii) DNA injection on a SeqA-mKate2 strain.** The protocol for DNA injection onto a strain expressing SeqA-mKate2 (LZ1387) was adapted from our previous work (55). An overnight culture of LZ1387 was diluted for 100-fold in LBM and grown to an OD_600_ of ∼0.4; then, CaCl_2_ was added to 5 mM. Purified P1LZ1914 phages were mixed with the cells to reach an MOI of 2, followed by incubation for 30 min at 30°C to trigger both phage adsorption and viral DNA injection. One microliter of the sample was placed on a 1-mm 1.5% phosphate-buffered saline (PBS) agarose pad resting on a small coverslip (18 by 18 mm; Fisher Scientific). After 1 to 2 min, a large coverslip (24 by 50 mm; Fisher Scientific) was gently overlaid, and the sample was imaged under the microscope at 30°C.

Cell images were taken at 24 stage positions in the phase-contrast (100-ms exposure for cell recognition), CFP (500-ms exposure for fluorescent phages), and far-red (100-ms exposure for SeqA-mKate2 foci) channels with 5 *z* stacks with a spacing of 300 nm.

**(iii) DNA injection on a TetR-mNeonGreen strain.** An overnight culture of LZ2001 was diluted 100-fold in EZ rich defined medium with 0.2% glucose and 10 mM MgSO_4_. For long cells with λKil induction, the overnight culture of LZ2001 with pBAD33-λ*kil* was diluted 100-fold in EZ rich defined medium with 30% LB and 10 mM MgSO_4_. ʟ-Arabinose was added to a final concentration of 0.05% when the OD_600_ approached 0.08. The cells were grown to an OD_600_ of ∼0.4; then, CaCl_2_ was added to 5 mM. Purified P1LZ1914 phages were mixed with the cells to reach an MOI of 2, followed by incubation for 30 min at 30°C to trigger both phage adsorption and viral DNA injection. One microliter of the sample was placed on a 1-mm 1.5% agarose pad (PBS for cell imaging, EZ rich with 0.2% glucose for time-lapse movie) resting on a small coverslip (18 by 18 mm; Fisher Scientific). After 1 to 2 min, a large coverslip (24 by 50 mm; Fisher Scientific) was gently overlaid, and the sample was imaged under the microscope at 30°C.

A series of 7 *z*-axis images with a spacing of 300 nm were taken through the CFP channel using a 500-ms exposure for fluorescent phages and the Green2#FISH channel using a 300-ms exposure for TetR-mNeonGreen-labeled DNA foci. Cells were imaged at multiple stage positions (typically 16) in each experiment. During the time-lapse movie, the sample was imaged in the phase-contrast (100-ms exposure for cell recognition) and Green2#FISH (300-ms exposure for DNA behavior) channels at intervals of 5 min until cell fate was visible (3 h in total).

### RNA FISH.

Different probes were synthesized to target different phage transcripts (3′ TEG-Amino; Biosearch Technologies). Probes targeting *c1*, *coi*, and *lxc* were designed as described in a previous study (60) and labeled with Cy5 (GE Healthcare no. PA15101), 6-carboxytetramethylrhodamine (TAMRA) (Biosearch Technologies, no. SMF-1001-5), and Alexa Fluor 488 (Thermo Fisher no. A20000), respectively (Fig. 3A). Probes are listed in Table S3.

10.1128/mBio.01013-21.10 TABLE S3 smFISH RNA probes. Download Table S3, DOCX file, 0.02 MB. Copyright © 2021 Zhang et al. 2021 Zhang et al. https://creativecommons.org/licenses/by/4.0/ This content is distributed under the terms of the Creative Commons Attribution 4.0 International license.

To perform RNA FISH on infection samples, the overnight host cells were diluted 100-fold in 65 ml of fresh LBM and grown at 37°C to an OD_600_ of ∼0.4. For the infections on Lxc-overexpressed cells IPTG was added at a final concentration of 0.2 mM in the cell culture (LZ1938) when the OD_600_ approached 0.1. Then, CaCl_2_ was added to 5 mM. After that, a 750-μl aliquot of cells was separated as a negative control without phages. The rest of the cell culture was mixed with the purified P1LZ1856 to reach MOI of 0.2, 1, and 5, and the mixture was incubated at 30°C (0 min). At each time point (10, 20, 30, 40, 50, 60, 80, and 100 min) during incubation, a 750-μl aliquot of the mixture was placed in a new 15-ml centrifuge tube with 830 μl of 37% formaldehyde. This tube was left to shake on a nutator for 30 min and then centrifuged at 400 × *g* for 8 min to pellet the cells. Details of fixation, permeabilization, and hybridization were presented in previous studies (60, 75). Briefly, after fixation, the cells were washed three times with 1× PBS. Subsequently, the cells were permeabilized by resuspension in 70% ethanol for 2 h at room temperature and centrifuged to collect the cells. The pellet was then resuspended in wash solution (40% formamide, 2× SSC [1× SSC is 0.15 M NaCl plus 0.015 M sodium citrate]), incubated for 5 min at room temperature, and pelleted again, after which they were ready for hybridization. The cells were then resuspended in 25 μl hybridization solution (40% formamide, 2× SSC, 1 mg ml^−1^ E. coli tRNA, 2 mM ribonucleoside-vanadyl complex, and 0.2 mg ml^−1^ bovine serum albumin [BSA]) with each set of probes reaching a final concentration of 1 μM. The samples were then incubated in a 30°C water bath overnight. The next day, the cells were washed three times using wash solution by incubating the cell pellet for 30 min in a 30°C water bath. After the final wash, the cells were resuspended in wash solution plus 10 μg ml^−1^ DAPI (4′,6-diamidino-2-phenylindole) and incubated for 10 min at room temperature. This suspension was then pelleted and resuspended in 2× SSC. The sample was then ready for imaging.

Two microliters of the cell suspension was pipetted onto the center of a 24- by 50-mm coverslip. A 1.5% PBS agarose pad was laid slowly on top of the cell suspension droplet with a razor blade. The pad was covered with an 18- by 18-mm coverslip. Under the microscope, images were taken in the phase-contrast (100 ms), Cy5 (700 ms, *c1*), Cy3 (1 s, *coi*), green fluorescent protein (GFP) (2 s, *lxc*), and DAPI (30 ms, DAPI) channels at different stage positions.

### DNA FISH.

For DNA FISH for P1 DNA replication detection, probes were produced by PCR amplifying ∼3 kb of P1 DNA (dnafish-P1-for and dnafish-P1-rev primer pair, listed in Table S2), using a phage lysate as the template, and treating the purified PCR product with a PromoFluor500-dUTP nick translation kit (PromoCell) to generate DNA-PromoFluor500 fragments ranging from 100 to 500 bp.

10.1128/mBio.01013-21.9 TABLE S2 Primers. Download Table S2, DOCX file, 0.02 MB. Copyright © 2021 Zhang et al. 2021 Zhang et al. https://creativecommons.org/licenses/by/4.0/ This content is distributed under the terms of the Creative Commons Attribution 4.0 International license.

To perform DNA FISH on infection samples, the overnight host cells were diluted 100-fold into 50 ml of fresh LBM and grown at 37°C to an OD_600_ of ∼0.4. Then CaCl_2_ was added to 5 mM. After that, a 5-ml aliquot of cells was separated as a negative control without phages and mixed with 550 μl of 37% formaldehyde for fixation. The rest of the cell culture was mixed with the purified P1LZ1856 to reach an MOI of 0.2, followed by incubation at 30°C. At each time point (30, 40, 50, and 60 min) during incubation, 5 ml of the mixture was transferred to a 15-ml centrifuge tube with 550 μl of 37% formaldehyde. This tube was left to shake on a nutator for 30 min and then centrifuged at 4,000 × *g* for 3 min to pellet the cells.

Details of fixation, permeabilization, and hybridization steps are detailed in reference 76. Briefly, the fixed cells were washed with 1 ml of ice-cold 1× PBS three times and resuspended in 1 ml of GTE solution (50 mM glucose, 20 mM Tris-HCl [pH 7.5], 10 mM EDTA). For the control sample, three separate 500-μl aliquots of the cell suspension were then mixed with 10 μl of 0.01 μg μl^−1^ lysozyme solution and incubated at room temperature for 2, 4, and 6 min, followed by three washes with GTE, and the cells were pelleted via centrifugation at 10,000 × *g* for 30 s. The cells were then resuspended in ∼150 μl of GTE. For each control sample, 1 μl of the cells was deposited onto a PBS agarose pad and imaged. The lysozyme treatment time yielding ∼90 to 95% intact cells (∼1 to 5% lysed cells) represents the optimal treatment time for the samples. The actual time point samples, from the initial GTE wash, were then processed as the control was, using the optimal lysozyme time. For each time point, 10 μl of cells were deposited onto poly-l-lysine-coated large coverslips (24 by 50 mm) and then covered with a smaller, normal coverslip (22 by 22 mm). The coverslips were then immersed in 1× PBS, and the smaller coverslip was removed, leaving only the sample coverslip. The cells were then dehydrated by immersing the coverslip in increasing concentrations of ethanol (70, 90, and100%). Samples were then ready for hybridization.

For each sample, approximately 160 μg of the probe mixture was combined with 10 μl of hybridization solution (50% formamide, 10% dextran sulfate, 50 mM NaPO_4_ [pH 7], 2× SSC). The double-stranded-DNA (dsDNA) probes were denatured at 75°C in a thermocycler and then placed on ice. Ten microliters of the denatured probe mixture was then deposited onto the center of the sample on the coverslip and overlaid with a small coverslip (22 by 22 mm). The small coverslip was then sealed with nail polish, forming a sample chamber. The chambers were incubated at 80°C for 5 min to denature the cellular DNA and then placed on Kimwipes over ice for 5 min. The chambers were then incubated in a 37°C incubator overnight to complete hybridization. The next day, the chambers were immersed in 2× SSC until the smaller coverslip dislodged. The remaining coverslips were soaked in wash solution (2× SSC, 50% formamide) for 20 min at 37°C twice. The coverslips were then washed with SSC in a series of increasing concentrations (1×, 2×, and 4×), each for 5 min at room temperature. A DAPI solution was then made by mixing 1 μl of 10 mg ml^−1^ DAPI with 1 ml of 4× SSC. For each sample, 500 μl of the DAPI solution was added over the sample, covering it, and incubated for 5 min at room temperature. After the coverslip was dried, 10 μl of 2× SSC was added over the sample and overlaid with a small coverslip (22 by 22 mm). The samples were then imaged.

Under the microscope, images were taken in phase-contrast (100 ms), GFP (300 ms), and DAPI (30 ms) channels at different stage positions.

### qPCR.

The overnight host cells were diluted 100-fold in fresh LBM and grown at 37°C. During cell growth, methanol was kept at −20°C for cell fixation. When the cell culture reached an OD_600_ of ∼0.4, CaCl_2_ was added to a final concentration of 5 mM. We then transferred 2 ml of cells to a 50-ml centrifuge tube and mixed them with 2 ml ice-cold methanol as the negative-control sample. The rest of the cell culture was mixed with purified P1LZ1856 to reach an MOI of ∼1 and incubated at 30°C (0 min). For each time point (0, 10, 20, 30, 40, 50, 60, 70, and 80 min), 2 ml of the reaction was aliquoted to 50-ml tubes, vortexed for 10 s, and then centrifuged at 4,000 × *g* for 3 min. After removing the supernatant, we resuspended the cell pellet with 2 ml fresh ice-cold LB solution, added 2 ml ice-cold methanol, and vortexed it for another 10 s. The mixture was centrifuged at 4,000 × *g* for 3 min. After removal of the supernatant, the cell pellet was kept at −20°C until DNA extraction using an UltraClean microbial DNA isolation kit (Mo Bio Laboratories no. 12224-50). The DNAs were then diluted and used for qPCR with primers targeting the phage DNA (qPCR-P1DNA-for/rev). The E. coli DNA number was used as a reference using primers targeting the *dxs* gene (dsx-for/rev) (77). Amplification was done using SYBR green PCR master mix (Applied Biosystems no. 4309155) with a 250 nM concentration of each primer (Table S2).

### Microscopy imaging.

Imaging was performed on a Nikon Eclipse Ti inverted epifluorescence microscope using a 100× objective (Plan Fluo; numerical aperture [NA], 1.40; oil immersion) with a 2.5× TV relay lens, within the cage of an incubator (InVivo Scientific) at 30°C, and acquired using a cooled electron-multiplying charge-coupled device (EMCCD) camera (IXON 897; Andor, Belfast, UK). Cells were imaged under phase contrast and the following fluorescent filter cubes (parameters are given as excitation filter wavelength and bandwidth; dichroic beam splitter wavelength; emission filter wavelength and bandwidth; company and product number): CFP (436 nm, 20; 455 nm; 480 nm, 40; Nikon, 96361), YFP (539 nm, 21; 556 nm; 576 nm, 31; Chroma no. 49309), far red (592 nm, 21; 610 nm; 630 nm, 30; Chroma no. 49310), DAPI (350 nm, 50; 400 nm; 460 nm, 50; Nikon no. 96310), Cy3 (545 nm, 30; 570 nm; 610 nm, 75; Nikon no. 96323), Cy5 (615 nm, 70; 660 nm; 700 nm, 75; Nikon no. 96366), and Green2#FISH (490 nm, 20; 505 nm; 525 nm, 30; Chroma no. 49308 [custom]). For microscopy images in our figures and movies, uniform contrast settings were applied for each separate channel throughout the figure subpanels or movie, unless otherwise stated.

### Data analysis.

**(i) Analysis of time-lapse movies.** Movie images were analyzed first using the model created by deep learning with TensorFlow for automatic cell recognition (collaboration with the lab of Anxiao Jiang, Department of Computer Science & Engineering, Texas A&M University) and then manually checked using the program Schnitzcells (gift from Michael Elowitz, California Institute of Technology), in order to generate a cellular index. The numbers of phages attached on cell surfaces, as well as distances between phages on the cells with an MOI of 2 and cell lengths, were measured manually using the supporting tools of the NIS-Elements program. Cell fates and DNA behavior for each infected cell was recorded as well. All subsequent data analysis was performed in MATLAB.

**(ii) “Failed” infections.** We observed that, in some cases, adsorbed phages were seen on the cell surface, but the cell grew normally with neither lysis nor lysogeny detected, similar to phage λ infection (41). These events were defined as “failed infections.” In our experiments, the rate of failed infection was defined as the number of failed infections divided by total number of infected cells with an MOI of 1. In the decision-making reporter system, the failed infection rate was 16.6% (99 of 596). In the SeqA-FP DNA visualization system, the failed infection rate was 21.6% (19 of 88). In the *tetO*/TetR-FP system, the failed infection frequency was 21.5% (31 of 144). A possible explanation for this phenomenon is the failure of the adsorbed phage to inject its DNA into the cell.

**(iii) “Dark” infections.** Similar to phage λ infection, a fraction of the cells exhibited dark infections: cells without any observed infecting phages on the cell surface exhibiting lysis or lysogeny. With careful treatment of the sample, the dark infection rate was ∼20% (120 cells at an MOI of 0 showing mVenus signal, compared to 596 cells at an MOI of 1), testing in the decision-making reporter system. The calculation is based on the assumption that dark infections were mainly MOI = 1 events. We hypothesize that these dark infections are mainly due to the phages falling off after injecting their DNA into the host cell. Another possibility is that some cells may divide into two daughter cells during the 30-min incubation, resulting in an infected daughter cell without an observed infecting phage. Similarly, in the SeqA-FP DNA visualization system, the dark infection rate was 15.9% (14 cells at an MOI of 0 showing DNA signal, compared to 88 cells at an MOI of 1), and in the *tetO*/TetR-FP system, the dark infection rate was 22.9% (33 cells at an MOI of 0 showing DNA signal, compared to 144 cells at an MOI of 1).

**(iv) Analysis of DNA number and RNA number.** Microscope images of the DNA injection in SeqA-FP or *tetO*/TetR-PF system and RNA FISH experiments were processed for cell recognition as described above. Then, the number of viral DNA molecules was analyzed via the procedure reported in reference 60. Basically, fluorescent spots were first identified from the stacks of fluorescence images, using the Spätzcells program. Then, false-positive spots, corresponding to the unspecific binding of probes to nontarget RNA, were identified by comparison with the negative control and discarded. After that, the spot intensity corresponding to a single mRNA molecule was identified by examining the single spot intensities in a low-expression sample (20 min), where individual mRNAs are spatially separable. Finally, the one-mRNA intensity value was used to convert the total spot intensity in cells from other samples to the number of target mRNA molecules.

**(v) Calculation of predicted intracellular DNA number due to failed and dark infection.** The frequencies of failed and dark phage infections at an MOI of 1 were designated *a* and *b*, respectively, and the frequencies of failed and dark infection for an infected cell are *a^n^* and *b^n^*, where *n* is the number of failed or dark phages. Here, we only considered a value for *n* of 1, since the frequencies are low when *n* is >1. Thus, there are four situations: none failed × none dark, none failed × one dark, one failed × one dark, and one failed × none dark. The predicted number of injected DNA for an MOI of 1 is calculated as

(1−a)×(1−b)+2×(1−a)×b+a×b

That for an MOI of 2 is

$$2\times {\left(1-a\right)}^{2}\times \left(1-b\right)+3\times {\left(1-a\right)}^{2}\times b+2\times 2\times \left(1-a\right)\times a\times b+2\times (1-a)\times a\times (1-b)$$

That for an MOI of 3 is

$$3\times {\left(1-a\right)}^{3}\times \left(1-b\right)+4\times {\left(1-a\right)}^{3}\times b+3\times 3\times {\left(1-a\right)}^{2}\times a\times b+2\times 3\times {\left(1-a\right)}^{2}\times a\times (1-b)$$

That for an MOI of 4 is

$$4\times {\left(1-a\right)}^{4}\times \left(1-b\right)+5\times {\left(1-a\right)}^{4}\times b+4\times 4\times {\left(1-a\right)}^{3}\times a\times b+3\times 4\times {\left(1-a\right)}^{3}\times a\times (1-b)$$

That for an MOI of 5 is

$$5\times {\left(1-a\right)}^{5}\times \left(1-b\right)+6\times {\left(1-a\right)}^{5}\times b+5\times 5\times {\left(1-a\right)}^{4}\times a\times b+4\times 5\times {\left(1-a\right)}^{4}\times a\times (1-b)$$

From our measurements, in the SeqA-FP system we found that *a* was 21.6% and *b* was 15.9%. Therefore, the sum probabilities of these four situations were 1, 0.95, 0.88, 0.79, and 0.70 for MOI from 1 to 5, respectively. The predictions of DNA number were 0.94, 1.72, 2.38, 2.89, and 3.22, compared with the quantified data (1.07, 1.70, 2.40, 2.57, and 3.80) using Spätzcells (60). In the *tetO*/TetR-FP system, *a* was 21.5% and *b* was 22.9%. Therefore, the sum probabilities of these four situations were 1, 0.95, 0.88, 0.80, and 0.71 for MOI from 1 to 4, respectively. The predictions of DNA number were 1.01, 1.79, 2.45, and 3.21, compared with the quantified data (0.93, 1.55, 2.28, and 3.30) using Spätzcells.

## References

[1] Balcazar JL. 2014. Bacteriophages as vehicles for antibiotic resistance genes in the environment. PLoS Pathog 10:e1004219. doi:10.1371/journal.ppat.1004219. 25078987 PMC4117541

[2] Billard-Pomares T, Fouteau S, Jacquet ME, Roche D, Barbe V, Castellanos M, Bouet JY, Cruveiller S, Medigue C, Blanco J, Clermont O, Denamur E, Branger C. 2014. Characterization of a P1-like bacteriophage carrying an SHV-2 extended-spectrum beta-lactamase from an Escherichia coli strain. Antimicrob Agents Chemother 58:6550–6557. doi:10.1128/AAC.03183-14. 25136025 PMC4249366

[3] Brown-Jaque M, Calero-Caceres W, Muniesa M. 2015. Transfer of antibiotic-resistance genes via phage-related mobile elements. Plasmid 79:1–7. doi:10.1016/j.plasmid.2015.01.001. 25597519

[4] Shin J, Ko KS. 2015. A plasmid bearing the bla(CTX-M-15) gene and phage P1-like sequences from a sequence type 11 Klebsiella pneumoniae isolate. Antimicrob Agents Chemother 59:6608–6610. doi:10.1128/AAC.00265-15. 26195513 PMC4576088

[5] Li R, Xie M, Lv J, Wai-Chi Chan E, Chen S. 2017. Complete genetic analysis of plasmids carrying mcr-1 and other resistance genes in an Escherichia coli isolate of animal origin. J Antimicrob Chemother 72:696–699. doi:10.1093/jac/dkw509. 27999050

[6] Yang L, Li W, Jiang GZ, Zhang WH, Ding HZ, Liu YH, Zeng ZL, Jiang HX. 2017. Characterization of a P1-like bacteriophage carrying CTX-M-27 in Salmonella spp. resistant to third generation cephalosporins isolated from pork in China. Sci Rep 7:40710. doi:10.1038/srep40710. 28098241 PMC5241659

[7] Brown-Jaque M, Calero-Caceres W, Espinal P, Rodriguez-Navarro J, Miro E, Gonzalez-Lopez JJ, Cornejo T, Hurtado JC, Navarro F, Muniesa M. 2018. Antibiotic resistance genes in phage particles isolated from human faeces and induced from clinical bacterial isolates. Int J Antimicrob Agents 51:434–442. doi:10.1016/j.ijantimicag.2017.11.014. 29180282

[8] Brown-Jaque M, Rodriguez Oyarzun L, Cornejo-Sanchez T, Martin-Gomez MT, Gartner S, de Gracia J, Rovira S, Alvarez A, Jofre J, Gonzalez-Lopez JJ, Muniesa M. 2018. Detection of bacteriophage particles containing antibiotic resistance genes in the sputum of cystic fibrosis patients. Front Microbiol 9:856. doi:10.3389/fmicb.2018.00856. 29765367 PMC5938348

[9] Venturini C, Zingali T, Wyrsch ER, Bowring B, Iredell J, Partridge SR, Djordjevic SP. 2019. Diversity of P1 phage-like elements in multidrug resistant Escherichia coli. Sci Rep 9:18861. doi:10.1038/s41598-019-54895-4. 31827120 PMC6906374

[10] Sweere JM, Van Belleghem JD, Ishak H, Bach MS, Popescu M, Sunkari V, Kaber G, Manasherob R, Suh GA, Cao X, de Vries CR, Lam DN, Marshall PL, Birukova M, Katznelson E, Lazzareschi DV, Balaji S, Keswani SG, Hawn TR, Secor PR, Bollyky PL. 2019. Bacteriophage trigger antiviral immunity and prevent clearance of bacterial infection. Science 363:eaat9691. doi:10.1126/science.aat9691. 30923196 PMC6656896

[11] Hendrickx APA, Landman F, de Haan A, Borst D, Witteveen S, van Santen-Verheuvel MG, van der Heide HGJ, Schouls LM, Dutch C, Dutch CPE Surveillance Study Group. 2020. Plasmid diversity among genetically related Klebsiella pneumoniae blaKPC-2 and blaKPC-3 isolates collected in the Dutch national surveillance. Sci Rep 10:16778. doi:10.1038/s41598-020-73440-2. 33033293 PMC7546619

[12] Kamal SM, Cimdins-Ahne A, Lee C, Li F, Martin-Rodriguez AJ, Seferbekova Z, Afasizhev R, Wami HT, Katikaridis P, Meins L, Lunsdorf H, Dobrindt U, Mogk A, Romling U. 2021. A recently isolated human commensal Escherichia coli ST10 clone member mediates enhanced thermotolerance and tetrathionate respiration on a P1 phage-derived IncY plasmid. Mol Microbiol 115:255–271. doi:10.1111/mmi.14614. 32985020 PMC7984374

[13] Bertani G. 1951. Studies on lysogenesis. I. The mode of phage liberation by lysogenic Escherichia coli. J Bacteriol 62:293–300. doi:10.1128/jb.62.3.293-300.1951. 14888646 PMC386127

[14] Kondo E, Mitsuhashi S. 1966. Drug resistance of enteric bacteria. VI. Introduction of bacteriophage P1CM into Salmonella typhi and formation of PldCM and F-CM elements. J Bacteriol 91:1787–1794. doi:10.1128/jb.91.5.1787-1794.1966. 5327907 PMC316124

[15] Okada M, Watanabe T. 1968. Transduction with phage P1 in Salmonella typhimurium. Nature 218:185–187. doi:10.1038/218185a0. 4868832

[16] Ornellas EP, Stocker BA. 1974. Relation of lipopolysaccharide character to P1 sensitivity in Salmonella typhimurium. Virology 60:491–502. doi:10.1016/0042-6822(74)90343-2. 4602344

[17] Murooka Y, Harada T. 1979. Expansion of the host range of coliphage P1 and gene transfer from enteric bacteria to other gram-negative bacteria. Appl Environ Microbiol 38:754–757. doi:10.1128/aem.38.4.754-757.1979. 395900 PMC243574

[18] Amati P. 1962. Abortive infection of Pseudomonas aeruginosa and Serratia marcescens with coliphage P1. J Bacteriol 83:433–434. doi:10.1128/jb.83.2.433-434.1962. 13860881 PMC277750

[19] Kaiser D, Dworkin M. 1975. Gene transfer to myxobacterium by Escherichia coli phage P1. Science 187:653–654. doi:10.1126/science.803710. 803710

[20] Yarmolinsky MB, Sternberg N. 1988. Bacteriophage P1, p 291–438. *In* Calendar R (ed), The bacteriophages. Plenum Press, New York, NY.

[21] Sandulache R, Prehm P, Kamp D. 1984. Cell wall receptor for bacteriophage Mu G(+). J Bacteriol 160:299–303. doi:10.1128/jb.160.1.299-303.1984. 6384194 PMC214716

[22] Calendar R, Calendar RL, Abedon ST. 2006. The Bacteriophages. Oxford University Press, New York, NY.

[23] Iida S, Streiff MB, Bickle TA, Arber W. 1987. Two DNA antirestriction systems of bacteriophage P1, darA, and darB: characterization of darA- phages. Virology 157:156–166. doi:10.1016/0042-6822(87)90324-2. 3029954

[24] Streiff MB, Iida S, Bickle TA. 1987. Expression and proteolytic processing of the darA antirestriction gene product of bacteriophage P1. Virology 157:167–171. doi:10.1016/0042-6822(87)90325-4. 3029955

[25] Iida S, Hiestand-Nauer R, Sandmeier H, Lehnherr H, Arber W. 1998. Accessory genes in the darA operon of bacteriophage P1 affect antirestriction function, generalized transduction, head morphogenesis, and host cell lysis. Virology 251:49–58. doi:10.1006/viro.1998.9405. 9813202

[26] Eliason JL, Sternberg N. 1987. Characterization of the binding sites of c1 repressor of bacteriophage P1. Evidence for multiple asymmetric sites. J Mol Biol 198:281–293. doi:10.1016/0022-2836(87)90313-5. 3430609

[27] Osborne FA, Stovall SR, Baumstark BR. 1989. The c1 genes of P1 and P7. Nucleic Acids Res 17:7671–7680. doi:10.1093/nar/17.19.7671. 2678003 PMC334876

[28] Łobocka MB, Rose DJ, Plunkett G, Rusin M, Samojedny A, Lehnherr H, Yarmolinsky MB, Blattner FR. 2004. Genome of bacteriophage P1. J Bacteriol 186:7032–7068. doi:10.1128/JB.186.21.7032-7068.2004. 15489417 PMC523184

[29] Baumstark BR, Stovall SR, Bralley P. 1990. The ImmC region of phage P1 codes for a gene whose product promotes lytic growth. Virology 179:217–227. doi:10.1016/0042-6822(90)90291-x. 2120849

[30] Heinzel T, Velleman M, Schuster H. 1990. The c1 repressor inactivator protein coi of bacteriophage P1. Cloning and expression of coi and its interference with c1 repressor function. J Biol Chem 265:17928–17934. doi:10.1016/S0021-9258(18)38252-8. 2211669

[31] Heinzel T, Velleman M, Schuster H. 1992. C1 repressor of phage P1 is inactivated by noncovalent binding of P1 Coi protein. J Biol Chem 267:4183–4188. doi:10.1016/S0021-9258(19)50646-9. 1740459

[32] Schaefer TS, Hays JB. 1990. The bof gene of bacteriophage P1: DNA sequence and evidence for roles in regulation of phage c1 and ref genes. J Bacteriol 172:3269–3277. doi:10.1128/jb.172.6.3269-3277.1990. 2345146 PMC209135

[33] Velleman M, Heirich M, Gunther A, Schuster H. 1990. A bacteriophage P1-encoded modulator protein affects the P1 c1 repression system. J Biol Chem 265:18511–18517. doi:10.1016/S0021-9258(17)44781-8. 2211715

[34] Velleman M, Heinzel T, Schuster H. 1992. The Bof protein of bacteriophage P1 exerts its modulating function by formation of a ternary complex with operator DNA and C1 repressor. J Biol Chem 267:12174–12181. doi:10.1016/S0021-9258(19)49820-7. 1601883

[35] Heinrich J, Velleman M, Schuster H. 1995. The tripartite immunity system of phages P1 and P7. FEMS Microbiol Rev 17:121–126. doi:10.1111/j.1574-6976.1995.tb00193.x. 7669337

[36] Dreiseikelmann B, Velleman M, Schuster H. 1988. The c1 repressor of bacteriophage P1. Isolation and characterization of the repressor protein. J Biol Chem 263:1391–1397. doi:10.1016/S0021-9258(19)57316-1. 2826478

[37] Citron M, Schuster H. 1992. The c4 repressor of bacteriophage P1 is a processed 77 base antisense RNA. Nucleic Acids Res 20:3085–3090. doi:10.1093/nar/20.12.3085. 1620606 PMC312442

[38] Heinrich J, Citron M, Gunther A, Schuster H. 1994. Second-site suppressors of the bacteriophage P1 virs mutant reveal the interdependence of the c4, icd, and ant genes in the P1 immI operon. J Bacteriol 176:4931–4936. doi:10.1128/jb.176.16.4931-4936.1994. 8051007 PMC196329

[39] Kourilsky P. 1973. Lysogenization by bacteriophage lambda. I. Multiple infection and the lysogenic response. Mol Gen Genet 122:183–195. doi:10.1007/BF00435190. 4573866

[40] Kourilsky P, Knapp A. 1974. Lysogenization by bacteriophage lambda. III. Multiplicity dependent phenomena occuring upon infection by lambda. Biochimie 56:1517–1523. 4619342

[41] Zeng L, Skinner SO, Zong C, Sippy J, Feiss M, Golding I. 2010. Decision making at a subcellular level determines the outcome of bacteriophage infection. Cell 141:682–691. doi:10.1016/j.cell.2010.03.034. 20478257 PMC2873970

[42] Silpe JE, Bassler BL. 2019. Phage-encoded LuxR-type receptors responsive to host-produced bacterial quorum-sensing autoinducers. mBio 10:e00638-19. doi:10.1128/mBio.00638-19. 30967469 PMC6456758

[43] Silpe JE, Bassler BL. 2019. A host-produced quorum-sensing autoinducer controls a phage lysis-lysogeny decision. Cell 176:268–280.E13. doi:10.1016/j.cell.2018.10.059. 30554875 PMC6329655

[44] Erez Z, Steinberger-Levy I, Shamir M, Doron S, Stokar-Avihail A, Peleg Y, Melamed S, Leavitt A, Savidor A, Albeck S, Amitai G, Sorek R. 2017. Communication between viruses guides lysis-lysogeny decisions. Nature 541:488–493. doi:10.1038/nature21049. 28099413 PMC5378303

[45] Stokar-Avihail A, Tal N, Erez Z, Lopatina A, Sorek R. 2019. Widespread utilization of peptide communication in phages infecting soil and pathogenic bacteria. Cell Host Microbe 25:746–755.E5. doi:10.1016/j.chom.2019.03.017. 31071296 PMC6986904

[46] Bertani G, Nice SJ. 1954. Studies on lysogenesis. II. The effect of temperature on the lysogenization of Shigella dysenteriae with phage P1. J Bacteriol 67:202–209. doi:10.1128/jb.67.2.202-209.1954. 13129214 PMC357203

[47] Rosner JL. 1972. Formation, induction, and curing of bacteriophage P1 lysogens. Virology 48:679–689. doi:10.1016/0042-6822(72)90152-3. 4555608

[48] Hadi SM, Bachi B, Iida S, Bickle TA. 1983. DNA restriction–modification enzymes of phage P1 and plasmid p15B. Subunit functions and structural homologies. J Mol Biol 165:19–34. doi:10.1016/S0022-2836(83)80240-X. 6302281

[49] Iida S, Meyer J, Bachi B, Stalhammar-Carlemalm M, Schrickel S, Bickle TA, Arber W. 1983. DNA restriction–modification genes of phage P1 and plasmid p15B. Structure and in vitro transcription. J Mol Biol 165:1–18. doi:10.1016/s0022-2836(83)80239-3. 6302279

[50] Humbelin M, Suri B, Rao DN, Hornby DP, Eberle H, Pripfl T, Kenel S, Bickle TA. 1988. Type III DNA restriction and modification systems EcoP1 and EcoP15. Nucleotide sequence of the EcoP1 operon, the EcoP15 mod gene and some EcoP1 mod mutants. J Mol Biol 200:23–29. doi:10.1016/0022-2836(88)90330-0. 2837577

[51] Ikeda H, Tomizawa J. 1968. Prophage P1, and extrachromosomal replication unit. Cold Spring Harbor Symp Quant Biol 33:791–798. doi:10.1101/sqb.1968.033.01.091. 4892009

[52] Prentki P, Chandler M, Caro L. 1977. Replication of prophage P1 during the cell cycle of Escherichia coli. Mol Gen Genet 152:71–76. doi:10.1007/BF00264942. 325389

[53] Lehnherr H, Maguin E, Jafri S, Yarmolinsky MB. 1993. Plasmid addiction genes of bacteriophage P1: doc, which causes cell death on curing of prophage, and phd, which prevents host death when prophage is retained. J Mol Biol 233:414–428. doi:10.1006/jmbi.1993.1521. 8411153

[54] Gazit E, Sauer RT. 1999. The Doc toxin and Phd antidote proteins of the bacteriophage P1 plasmid addiction system form a heterotrimeric complex. J Biol Chem 274:16813–16818. doi:10.1074/jbc.274.24.16813. 10358024

[55] Zhang K, Young R, Zeng L. 2020. Bacteriophage P1 does not show spatial preference when infecting Escherichia coli. Virology 542:1–7. doi:10.1016/j.virol.2019.12.012. 31957661 PMC7024032

[56] Devlin BH, Baumstark BR, Scott JR. 1982. Superimmunity: characterization of a new gene in the immunity region of P1. Virology 120:360–375. doi:10.1016/0042-6822(82)90037-x. 6285609

[57] Kliem M, Dreiseikelmann B. 1989. The superimmunity gene sim of bacteriophage P1 causes superinfection exclusion. Virology 171:350–355. doi:10.1016/0042-6822(89)90602-8. 2763457

[58] Shao Q, Hawkins A, Zeng L. 2015. Phage DNA dynamics in cells with different fates. Biophys J 108:2048–2060. doi:10.1016/j.bpj.2015.03.027. 25902444 PMC4407255

[59] Trinh JT, Szekely T, Shao Q, Balazsi G, Zeng L. 2017. Cell fate decisions emerge as phages cooperate or compete inside their host. Nat Commun 8:14341. doi:10.1038/ncomms14341. 28165024 PMC5303824

[60] Skinner SO, Sepulveda LA, Xu H, Golding I. 2013. Measuring mRNA copy number in individual Escherichia coli cells using single-molecule fluorescent in situ hybridization. Nat Protoc 8:1100–1113. doi:10.1038/nprot.2013.066. 23680982 PMC4029592

[61] Trinh JT, Shao Q, Guan J, Zeng L. 2020. Emerging heterogeneous compartments by viruses in single bacterial cells. Nat Commun 11:3813. doi:10.1038/s41467-020-17515-8. 32732913 PMC7393140

[62] Shao Q, Trinh JT, McIntosh CS, Christenson B, Balazsi G, Zeng L. 2017. Lysis-lysogeny coexistence: prophage integration during lytic development. Microbiologyopen 6:e00395. doi:10.1002/mbo3.395. PMC5300877 27530202

[63] Lau IF, Filipe SR, Soballe B, Okstad OA, Barre FX, Sherratt DJ. 2003. Spatial and temporal organization of replicating Escherichia coli chromosomes. Mol Microbiol 49:731–743. doi:10.1046/j.1365-2958.2003.03640.x. 12864855

[64] Piya D, Vara L, Russell WK, Young R, Gill JJ. 2017. The multicomponent antirestriction system of phage P1 is linked to capsid morphogenesis. Mol Microbiol 105:399–412. doi:10.1111/mmi.13705. 28509398 PMC6011833

[65] Greer H. 1975. The kil gene of bacteriophage lambda. Virology 66:589–604. doi:10.1016/0042-6822(75)90231-7. 1098278

[66] Haeusser DP, Hoashi M, Weaver A, Brown N, Pan J, Sawitzke JA, Thomason LC, Court DL, Margolin W. 2014. The Kil peptide of bacteriophage lambda blocks Escherichia coli cytokinesis via ZipA-dependent inhibition of FtsZ assembly. PLoS Genet 10:e1004217. doi:10.1371/journal.pgen.1004217. 24651041 PMC3961180

[67] Romantsov T, Helbig S, Culham DE, Gill C, Stalker L, Wood JM. 2007. Cardiolipin promotes polar localization of osmosensory transporter ProP in Escherichia coli. Mol Microbiol 64:1455–1465. doi:10.1111/j.1365-2958.2007.05727.x. 17504273

[68] Bakshi S, Choi H, Weisshaar JC. 2015. The spatial biology of transcription and translation in rapidly growing Escherichia coli. Front Microbiol 6:636. doi:10.3389/fmicb.2015.00636. 26191045 PMC4488752

[69] Rothenberg E, Sepulveda LA, Skinner SO, Zeng L, Selvin PR, Golding I. 2011. Single-virus tracking reveals a spatial receptor-dependent search mechanism. Biophys J 100:2875–2882. doi:10.1016/j.bpj.2011.05.014. 21689520 PMC3123979

[70] Edgar R, Rokney A, Feeney M, Semsey S, Kessel M, Goldberg MB, Adhya S, Oppenheim AB. 2008. Bacteriophage infection is targeted to cellular poles. Mol Microbiol 68:1107–1116. doi:10.1111/j.1365-2958.2008.06205.x. 18363799 PMC3740151

[71] Shao Q, Trinh JT, Zeng L. 2019. High-resolution studies of lysis-lysogeny decision-making in bacteriophage lambda. J Biol Chem 294:3343–3349. doi:10.1074/jbc.TM118.003209. 30242122 PMC6416446

[72] Bryant JA, Sellars LE, Busby SJ, Lee DJ. 2014. Chromosome position effects on gene expression in Escherichia coli K-12. Nucleic Acids Res 42:11383–11392. doi:10.1093/nar/gku828. 25209233 PMC4191405

[73] Neidhardt FC, Bloch PL, Smith DF. 1974. Culture medium for enterobacteria. J Bacteriol 119:736–747. doi:10.1128/jb.119.3.736-747.1974. 4604283 PMC245675

[74] Datsenko KA, Wanner BL. 2000. One-step inactivation of chromosomal genes in Escherichia coli K-12 using PCR products. Proc Natl Acad Sci USA 97:6640–6645. doi:10.1073/pnas.120163297. 10829079 PMC18686

[75] Shao Q, Cortes MG, Trinh JT, Guan J, Balazsi G, Zeng L. 2018. Coupling of DNA replication and negative feedback controls gene expression for cell-fate decisions. iScience 6:1–12. doi:10.1016/j.isci.2018.07.006. 30240603 PMC6137276

[76] Bates D, Kleckner N. 2005. Chromosome and replisome dynamics in E. coli: loss of sister cohesion triggers global chromosome movement and mediates chromosome segregation. Cell 121:899–911. doi:10.1016/j.cell.2005.04.013. 15960977 PMC2973560

[77] Lee C, Kim J, Shin SG, Hwang S. 2006. Absolute and relative QPCR quantification of plasmid copy number in Escherichia coli. J Biotechnol 123:273–280. doi:10.1016/j.jbiotec.2005.11.014. 16388869

