# Supplementary figures and images for: Interactions between Viral Regulatory Proteins Ensure an MOI-Independent Probability of Lysogeny during Infection by Bacteriophage P1
Source: mBio. 2021 Sep 14;12(5):e01013-21. doi: 10.1128/mBio.01013-21 (PMC8546580; doi:10.1128/mBio.01013-21)

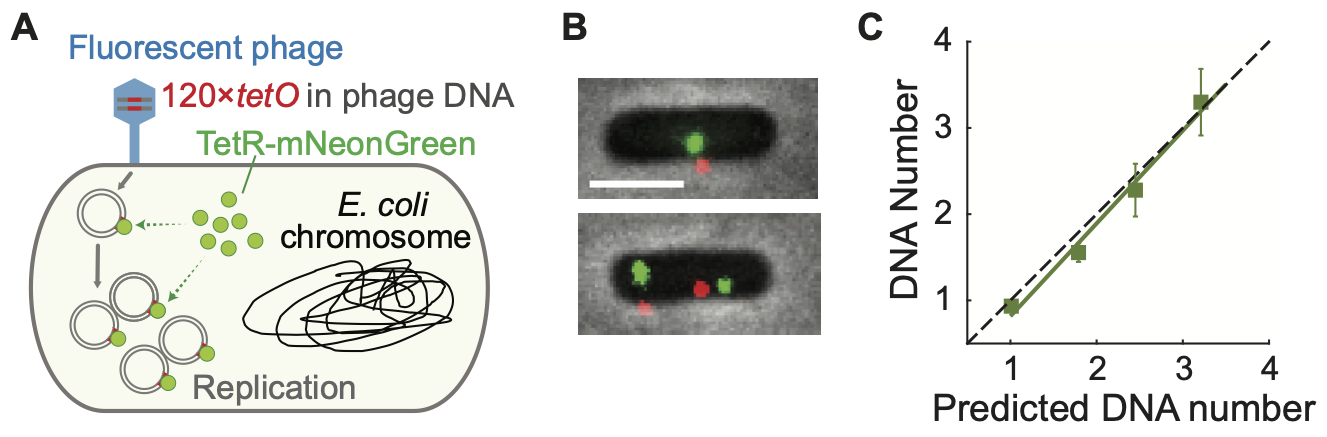

Supplement: FIG S2 [file mbio.01013-21-sf002.tif]

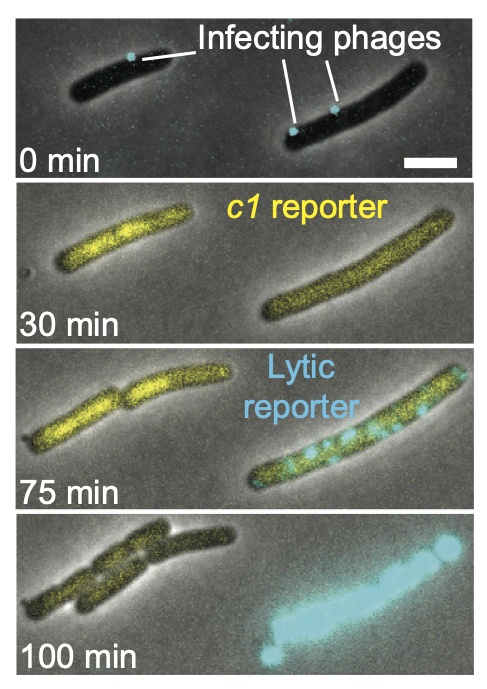

Supplement: FIG S1 [file mbio.01013-21-sf001.tif]

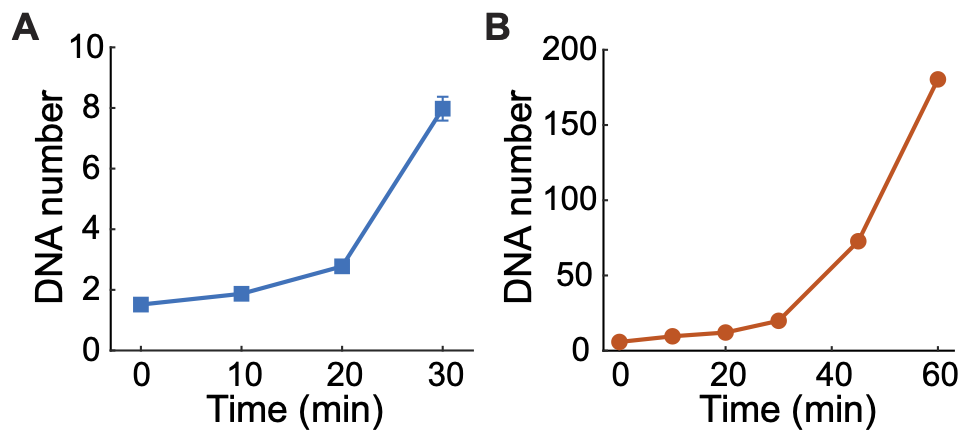

Supplement: FIG S3 [file mbio.01013-21-sf003.tif]

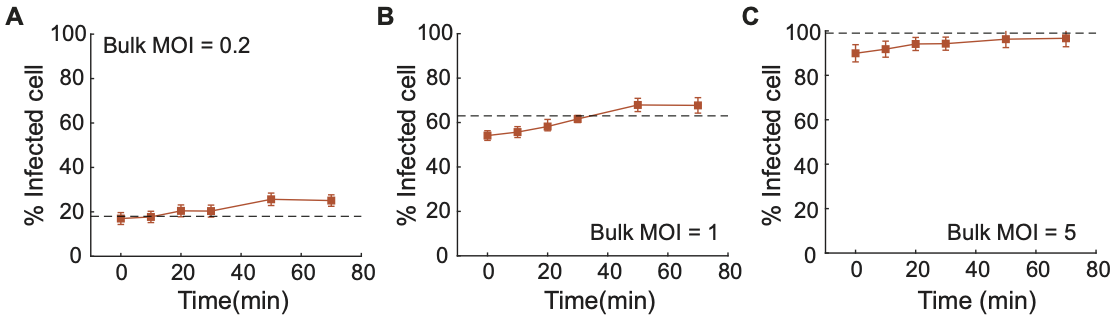

Supplement: FIG S4 [file mbio.01013-21-sf004.tif]

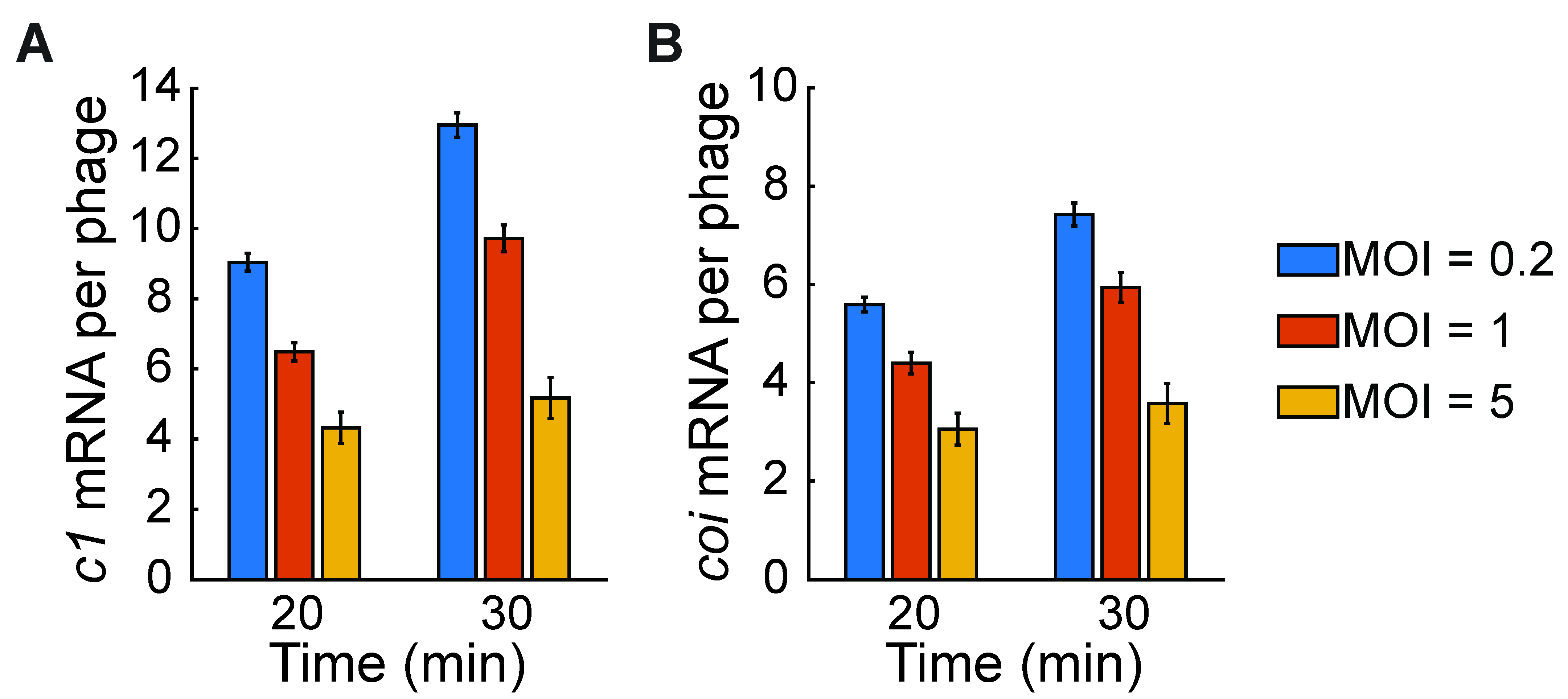

Supplement: FIG S5 [file mbio.01013-21-sf005.tif]

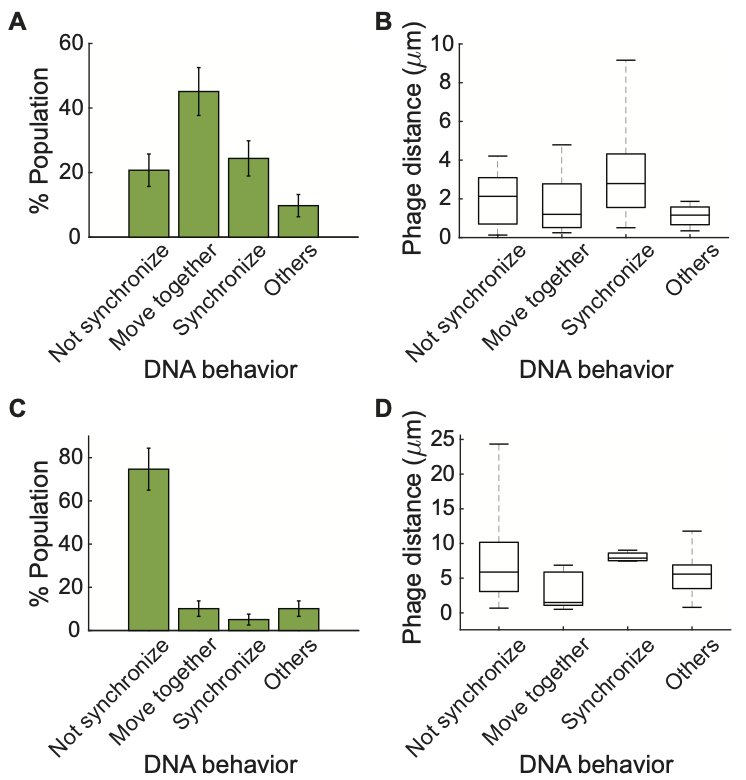

Supplement: FIG S6 [file mbio.01013-21-sf006.tif]

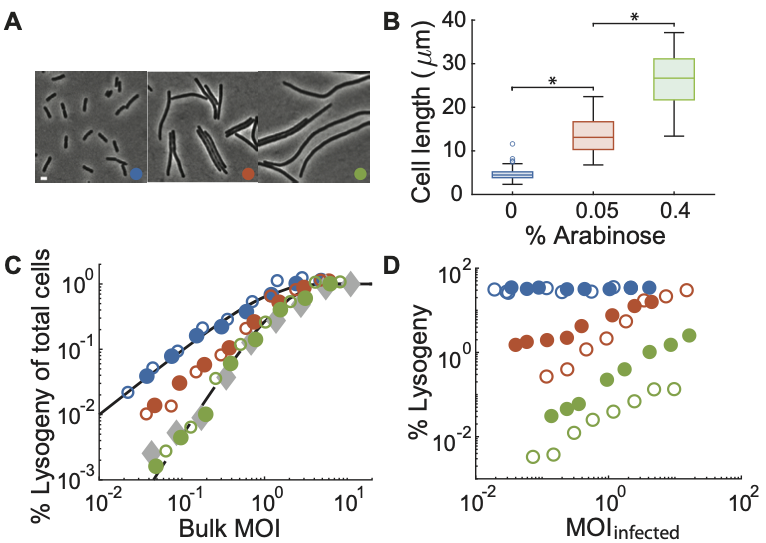

Supplement: FIG S7 [file mbio.01013-21-sf007.tif]
