# Supplementary material for: Interactions between Viral Regulatory Proteins Ensure an MOI-Independent Probability of Lysogeny during Infection by Bacteriophage P1
Source: mBio. 2021 Sep 14;12(5):e01013-21. doi: 10.1128/mBio.01013-21 (PMC8546580; doi:10.1128/mBio.01013-21)
Supplement: TABLE S1 [file mbio.01013-21-st001.docx]

**Table S1. Bacterial strains, phages, and plasmids**

| **Strain #** | **Genotype or relevant characteristic** | **Comments** | **Reference or Source** |
| --- | --- | --- | --- |
| MG1655 | F-, *lambda^-^, rph-1* | Wild type *E. coli*; indicator strain of phage titration and lysogenization in bulk; host stain in smFISH assays | Lab stock |
| LZ1387 | MG1655 *seqA-mKate2, Cm^R^-Frt, Δdam::Kan^R^* | Strain with SeqA reporter, *dam* deletion | ref. (1) |
| LZ1914 | MG1655 *lacIq, lacY^-^,* P1*Cm*, *c1*-100 *∆cin*, *c1-RBS-mVenus-Kan^R^* [pACYC177 PLlacO-1-*gene23-mTurquoise2*] | Strain to produce gp23-mTurquoise2 *c1-*labeled phages | ref. (2) |
| LZ1915 | MG1655 [pBR322 LP23*-gene23-mTurquoise2*] | Host strain with lytic reporter plasmid for single-cell infection movies | ref. (2) |
| LZ1931 | MG1655 *lacIq, lacY^-^,* [pZE12luc PLlacO-1-*lxc*] | Lxc overexpression strain used in bulk lysogenization assay | This work |
| LZ1937 | MG1655 *lacIq, lacY^-^,* [pACYC177 PLlacO-1-*lxc*] [pBR322 LP23*-gene23-mTurquoise2*] | Lxc overexpression strain used in single-cell infection movies | This work |
| LZ1938 | MG1655 *lacIq, lacY^-^,* [pACYC177 PLlacO-1-*lxc*] | Lxc overexpression strain used in smFISH assay | This work |
| LZ1952 | MG1655 [pBR322 LP23*-gene23-mTurquoise2*] [pACYC177 C1operator] | Host cells with extra C1 operators for single-cell infection movies | This work |
| LZ2001 | MG1655 [pZS24 PftsKi-*tetR-mNeonGreen*] | Host strain of *tetO/*TetR-FP DNA visualization system | Lab stock |
| LZ2500 | MG1655 [pBAD24-λ*kil*] | Longer cell strain | Gift from Karthik Chamakura and Ry Young |
| LZ2504 | MG1655 *lacIq, lacY^-^,* P1*Cm*, *c1*-100 *∆cin*, *darB::120tetO-Kan^R^* [pACYC177 PLlacO-1-*gene23-mTurquoise2*] | Strain to produce gp23-mTurquoise2 120×*tetO* phages | This work |
| **Phage** |  |  |  |
| P1LZ1856 | P1*Cm*, *c1-*100, *∆cin* | Phage with fixed tail fibers | This work |
| P1LZ1914 | P1*Cm*, *c1-*100, *∆cin, c1-*RBS*-mVenus-Kan^R^* | gp23-mTurquoise2 *c1-*labeled phage | This work |
| P1LZ2504 | P1*Cm*, *c1*-100 *∆cin*, *darB::120tetO-Kan^R^* | gp23-mTurquoise2 *tetO* phage | This work |
| **Plasmid** |  |  |  |
| pLZ1903 | pACYC177 PLlacO-1-*gene23-mTurquoise2* | Provides fluorescently labeled gp23 during lysogen induction to form fluorescent phages | This work |
| pKD46 |  | Temperature sensitive plasmid which inserts DNA using homologous recombination | Gift from Jason Gill |
| pLZ1915 | pBR322 LP23-*gene23-Turquoise2* | Lytic reporter plasmid | This work |
| pLZ1931 | pZE12luc PLlacO-1-*lxc* | Lxc expression under the control of IPTG | This work |
| pLZ1933 | pACYC177 PLlacO-1-*lxc* | Lxc expression under the control of IPTG | This work |
| pLZ1950 | pACYC177 C1operator | Plasmid with one copy of the consensus C1 operator sequence | This work |
| pLZ1981 | pUC19 *H1-proKan^R^-120tetO-H2* | The recombination plasmid to replace part of the *darB* region of P1KL1856 with *tetO* arrays. | This work |

**Supplemental references**

1. Trinh JT, Szekely T, Shao Q, Balazsi G, Zeng L. 2017. Cell fate decisions emerge as phages cooperate or compete inside their host. Nat Commun 8:14341.

2. Zhang K, Young R, Zeng L. 2020. Bacteriophage P1 does not show spatial preference when infecting Escherichia coli. Virology 542:1-7.
