# Supplementary material for: Interactions between Viral Regulatory Proteins Ensure an MOI-Independent Probability of Lysogeny during Infection by Bacteriophage P1
Source: mBio. 2021 Sep 14;12(5):e01013-21. doi: 10.1128/mBio.01013-21 (PMC8546580; doi:10.1128/mBio.01013-21)
Supplement: TABLE S3 [file mbio.01013-21-st003.docx]

**Table S3. smFISH RNA probes**

| Probe name | Sequence (5' to 3') |
| --- | --- |
| P1-coi-1 | gtattacgaaacggcggc |
| P1-coi-2 | aaatagtgaatccaaagt |
| P1-coi-3 | acaacgctgaagacacat |
| P1-coi-4 | catttccttctgagccgc |
| P1-coi-5 | taaggtttccctgtttgc |
| P1-coi-6 | tagctgtaatgccgttgt |
| P1-coi-7 | gtgtaagatgagcaatgt |
| P1-coi-8 | tctaacaacattgcgctg |
| P1-coi-9 | atgatccatgctggggta |
| P1-coi-10 | ctgacctactgtttcacc |
| P1-coi-11 | caacacagagcctgaagc |
| P1-coi-12 | ttatgcctcactgtattg |
| P1-coi-13 | ggtggaatgaaagccata |
| P1-coi-14 | tctaacgtcgtcgatggt |
| P1-coi-15 | ataaagcgttagagcaat |
| P1-coi-16 | gtttcggcggggtctaca |
| P1-coi-17 | aatggcgcgggcagcgtc |
| P1-coi-18 | tctttgagtagtgttcag |
| P1-coi-19 | cggtactcctgattggat |
| P1-coi-20 | caggtcgtcttgggtgat |
| P1-coi-21 | ttgtgtcagtgagatcat |
| P1-coi-22 | gtggccatgagatattcg |
| P1-coi-23 | ttgtgagtctggctggtt |
| P1-c1-1 | ggattacactgggagttgtt |
| P1-c1-2 | ttgacatggccaacaactca |
| P1-c1-3 | atttatcattgatcctcctc |
| P1-c1-4 | tacagttgttcgccgtagac |
| P1-c1-5 | aagagatccctgaagctgac |
| P1-c1-6 | gcgtgcaacagcttttttta |
| P1-c1-7 | aaccgggcgaacaggacgac |
| P1-c1-8 | gtttctttgaacggcagaac |
| P1-c1-9 | aatttcagcctgaatgctgt |
| P1-c1-10 | gcgccattaatgtccattta |
| P1-c1-11 | tgggatattgagatctgggt |
| P1-c1-12 | gcgaaggatgttaggtacag |
| P1-c1-13 | aagtcgtaaccttacgcaca |
| P1-c1-14 | gtcatattgacgctgttcac |

**Table S3. Continued**

| P1-c1-15 | tcagatgaataatgcggcca |
| --- | --- |
| P1-c1-16 | tggatgcgaatgtccttatc |
| P1-c1-17 | ttcgcactgtgctcattgat |
| P1-c1-18 | ttgcttccaggttctctatg |
| P1-c1-19 | tactgcttcattagctcttt |
| P1-c1-20 | tacggaatttttcatcctcc |
| P1-c1-21 | tttcgctaaagccgtgtacg |
| P1-c1-22 | atgtaatggacgcgcagcat |
| P1-c1-23 | attgtagttagggctactgg |
| P1-c1-24 | ggtaactaactgatttgcca |
| P1-c1-25 | aaacacgcaacatagcggca |
| P1-c1-26 | ctcggagagtttcatcgcag |
| P1-c1-27 | tcaccgttgatgatgattcc |
| P1-c1-28 | ggctaaattttgctttctca |
| P1-c1-29 | cgcagatgatcggttctata |
| P1-c1-30 | cgatatatttttgcctgcgg |
| P1-c1-31 | tacattttcaatatctgcca |
| P1-c1-32 | ttttctcttcgcgagtgatg |
| P1-c1-33 | aactgatgcggctgatttga |
| P1-c1-34 | ttcttagttttgcggctgcg |
| P1-c1-35 | ttcggggttgtcgtttactg |
| P1-lxc-1 | ccaaggcttcaacttcca |
| P1-lxc-2 | caagttttctctccagcc |
| P1-lxc-3 | cccatgctttactgtgta |
| P1-lxc-4 | tcttgtaatgctcgtagc |
| P1-lxc-5 | gcacctcaacgttatgct |
| P1-lxc-6 | agctttacttcccccttc |
| P1-lxc-7 | tttcccgtccggacggta |
| P1-lxc-8 | ttgaaatcgacgaccgtc |
| P1-lxc-9 | cctggggaacactgtttg |
| P1-lxc-10 | ttcgaatgcccggtcacg |
| P1-lxc-11 | atgatgatctgctcccat |
| P1-lxc-12 | cgcattgagaagcaatgc |
| P1-lxc-13 | ggtgagcaaacagccata |
| P1-lxc-14 | tcgtgcttctttgagcga |
| P1-lxc-15 | gtggcactatcactatct |
| P1-lxc-16 | agcccgttagagccagaa |
| P1-lxc-17 | ttagcaacaaagcgccgc |

**Table S3. Continued**

| P1-lxc-18 | tccattttaaaccagatc |
| --- | --- |
| P1-lxc-19 | ccgtttctacttcggaaa |
| P1-lxc-20 | gctttcaacacagccagt |
| P1-lxc-21 | ttatcaaaacactgtggg |
| P1-lxc-22 | ttcttgaatgaaggctcc |
| P1-lxc-23 | ctaataatctcaagcaat |
| P1-lxc-24 | agagttcaacctccgatg |
| P1-lxc-25 | gccagtttaaactgtatg |
