# Supplementary material for: Interactions between Viral Regulatory Proteins Ensure an MOI-Independent Probability of Lysogeny during Infection by Bacteriophage P1
Source: mBio. 2021 Sep 14;12(5):e01013-21. doi: 10.1128/mBio.01013-21 (PMC8546580; doi:10.1128/mBio.01013-21)
Supplement: TABLE S2 [file mbio.01013-21-st002.docx]

**Table S2. Primers**

| **Primer** | **Sequence** |
| --- | --- |
| 1 | GCGACCATGGGATTGAAAAAGCGATACTACACAGTAAAGC |
| 2 | TGCAGCATGCGGTGAGCAAACAGCCATAATTTG |
| 3 | CATGGGATCCCTCGAGAATTGTGAGCGGATAAC |
| 4 | CATGCTGCAGGTATTACCGCCTTTGAGTGAGC |
| 5 | GCGAGGATCCATTGCTCTAATAAATTTTAGAGCGCACGAATGAGGGC |
| 6 | ATTACTGCAGGTATCACGAGGCCCTTTCGTCTTC |
| 7 | ATTGTACTGAGAGTGCACCATATGGATGTTGCCAGTTATGGC |
| 8 | CAAGGGTACCGATCGCGAGTGAGATCAC |
| 9 | ACTCGCGATCGGTACCCTTGCAGTGGGCTTACATG |
| 10 | ATAGAGACTCGAGGGATCCGAATTCTCAGAAGAACTCGTCAAGAAGG |
| 11 | ACGTGTCGACCACCAATGCCATTTTCGGTACCC |
| 12 | TGTCAAGCTTAAGGGCCAGGTTTATCCGATCAG |
| dnafish-P1-for | CAACGAACCAGGTAGCCGGAATGTG |
| dnafish-P1-rev | AGCACAACCATCAACCAGCGCCAAA |
| qPCR-P1DNA-for | CTCTGCCCGTTATTTGTGGTGT |
| qPCR-P1DNA-rev | AGTCCGCTCGCTCTGTGTAG |
| dxs-for | CGAGAAACTGGCGATCCTTA |
| dxs-rev | CTTCATCAAGCGGTTTCACA |
